# Tuning Power Ultrasound for Enhanced Performance of Thermoplastic Micro-Injection Molding: Principles, Methods, and Performances

**DOI:** 10.3390/polym13172877

**Published:** 2021-08-27

**Authors:** Baishun Zhao, Yuanbao Qiang, Wangqing Wu, Bingyan Jiang

**Affiliations:** 1 State Key Laboratory of High-Performance Complex Manufacturing, Central South University, Lushan South Road 932, Changsha 410083, China; qustzbs@163.com (B.Z.); yuanbaoqiang@csu.edu.cn (Y.Q.); jby@csu.edu.cn (B.J.); 2 School of Mechanical and Electrical Engineering, Central South University, Lushan South Road 932, Changsha 410083, China

**Keywords:** micro-injection molding, ultrasonic molding, ultrasonic injection molding, ultrasonic micro-injection molding, power ultrasound, ultrasonic plasticization, ultrasonic vibration

## Abstract

With the wide application of Micro-Electro-Mechanical Systems (MEMSs), especially the rapid development of wearable flexible electronics technology, the efficient production of micro-parts with thermoplastic polymers will be the core technology of the harvesting market. However, it is significantly restrained by the limitations of the traditional micro-injection-molding (MIM) process, such as replication fidelity, material utilization, and energy consumption. Currently, the increasing investigation has been focused on the ultrasonic-assisted micro-injection molding (UAMIM) and ultrasonic plasticization micro-injection molding (UPMIM), which has the advantages of new plasticization principle, high replication fidelity, and cost-effectiveness. The aim of this review is to present the latest research activities on the action mechanism of power ultrasound in various polymer micro-molding processes. At the beginning of this review, the physical changes, chemical changes, and morphological evolution mechanism of various thermoplastic polymers under different application modes of ultrasonic energy field are introduced. Subsequently, the process principles, characteristics, and latest developments of UAMIM and UPMIM are scientifically summarized. Particularly, some representative performance advantages of different polymers based on ultrasonic plasticization are further exemplified with a deeper understanding of polymer–MIM relationships. Finally, the challenges and opportunities of power ultrasound in MIM are prospected, such as the mechanism understanding and commercial application.

## 1. Introduction

In the last decade and even in the future, some electromechanical systems with special functions and personal electronic products will continue to maintain the trend of miniaturization and precision. Therefore, the high-quality mass production of micro-parts has attracted significant attention. From the perspective of polymer processing technology, the advantages of thermoplastic micro-injection molding (MIM) are reflected in the short production cycle, large scale, good dimensional accuracy, and low restrictions on complex shapes and details [1]. Compared with other molding technologies, MIM is more suitable for low-cost mass production. Especially with the molding precision reaching nano-scale, MIM is believed to be the technology that could meet the needs of most micro–nano products on the market [2]. For instance, the smallest part in the world was fabricated by the MTD micro-molding company, weighing only 0.00313 mg, and then, Holzer et al. [3] successfully fabricated a part with nano grooves of 18 nm width. Although some researchers have shared their definitions of micro-molded products, which will be discussed later in this paper [1,4,5,6,7,8], the size of the micro-molded parts is expected to exceed their definitions due to the continuous development of MIM. In fact, micro-molded parts have been gradually extending to sub-micron [9,10,11] or even nano-scale [12,13,14].

Generally, micro-molded parts can be divided into two groups. One of them is macro-parts with cross-scale features such as micro/nano structures on functional surfaces [15,16,17]. The other is small parts with weight in milligram scale. Due to the limitations of the process characteristics and material properties, when the micro-molded parts comprise cross-scale features or break through a certain volume/size boundary, MIM could be quite challenging in terms of replication fidelity, materials utilization, and energy consumption. In this context, power ultrasound was introduced to enhance the MIM performance. Specifically, for the macro-parts with micro/nano features, the power ultrasound system has been integrated into the injection mold to facilitate the polymer melt filling and the replication of the micro structures [18]. For the small parts with milligram weight, power ultrasound has been employed as the only energy source during MIM, [19,20,21,22] where a small quantity of plastic raw materials can be injection molded directly after plasticization by ultrasonic vibration, without screw shearing and external heating.

Many researchers have studied the application of power ultrasound in the MIM of thermoplastic polymers in various aspects such as tooling, process modeling and simulation, and the micro-molded parts characterization. From the author’s point of view, these contributions followed different processing strategies and evolved into two typical MIM variants, i.e., ultrasound-assisted micro-injection molding (UAMIM) and ultrasonic plasticization micro-injection molding (UPMIM). Among their many differences, the main difference between them is whether to use the traditional screw unit or the power ultrasound to plasticize the plastic raw materials. Nevertheless, what they still have in common is both of them focus on tuning power ultrasound for enhanced MIM performance.

The purpose of this work is to share a detailed overview of the application status and development potential of tuning power ultrasound for the enhanced performance of thermoplastic MIM. The physical changes, chemical changes, and morphological evolution mechanism of various thermoplastic polymers under different application modes of ultrasonic energy field are introduced. Subsequently, the process principles, characteristics, and latest developments of UAMIM and UPMIM are summarized, which have not been found in the literature. Particularly, some representative performance advantages of different polymers based on ultrasonic plasticization are further exemplified with a deeper understanding of polymer–MIM relationships. Presently, both of the two MIM variants are in a critical period that requires rapid development and breakthrough results.

## 2. Ultrasound-Assisted Micro-Injection Molding

### 2.1. Scale Effect in MIM

**Figure 1:**
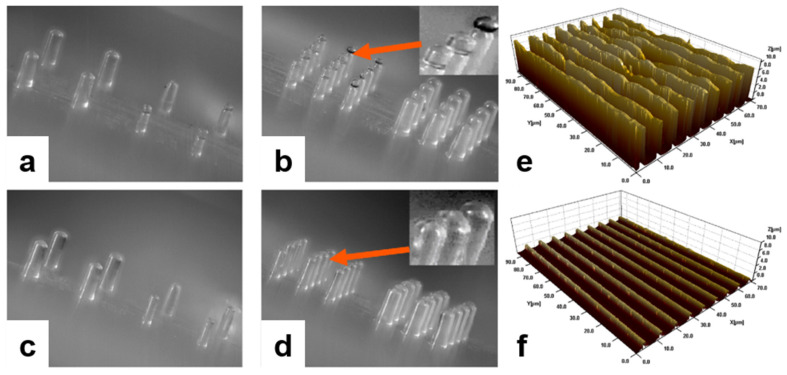
Microstructures molding by MIM. (**a**–**d**): Micro pins of PP [24]. (a and b): T_b_ = 225 °C, V_i_ = 100 mm/s, (**c**,**d**): T_b_ = 225 °C, V_i_ = 200 mm/s. T_b_ is the barrel temperature, V_i_ is the injection speed; Replicated micro features located at (**e**) 1.5 mm and (**f**) 35 mm from the gate [25].

High surface-to-volume ratios generally are the main feature of micro/nano-parts; thus, the polymer melt solidifies particularly rapidly due to the significant heat diffusion effects in the filling stage [22,23]. A formed solid layer can be a resistance to the melt injection, which is the main reason why filling defects such as uncompleted features exist. In the experiment of Sha et al. [24], the micro-needle cavities with a diameter of 100 μm and 150 μm could not be completely filled with melt under the conditions of low temperature and low injection speed, but when these parameters were set higher, the cavities could be completely filled (Figure 1a,d). The filling can also be complex and unstable in a smaller scale; it can be an increasingly difficult to fill into the cavity from the gate to the end of the mold insert. As shown in Figure 1e,f, filling height quickly decreases as the distance from the gate increases [25]. Therefore, the size effect poses a challenge to the high-quality forming of micro-scale features, which cannot be solved perfectly by optimizing process parameters.

**Figure 2:**
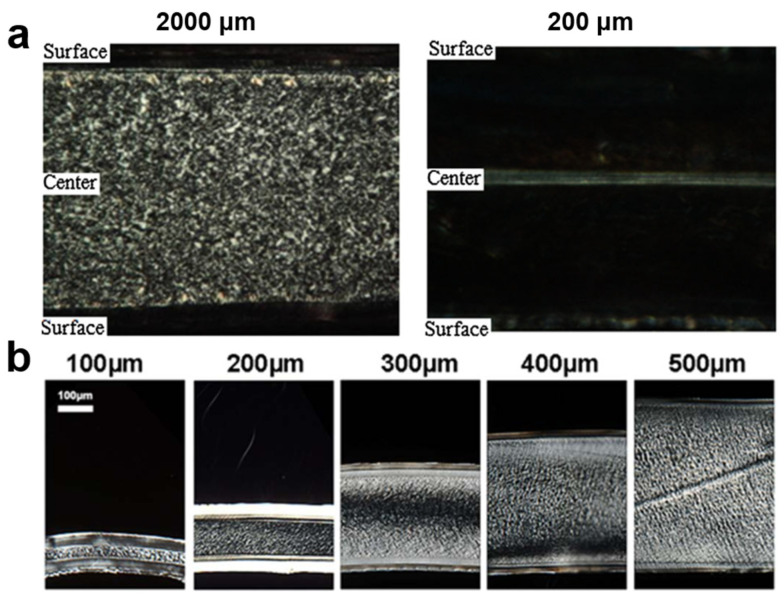
Morphology of “skin–core” structure in different scale. (**a**) Thickness from 2000 to 200 μm [28]; (**b**) Thickness from 100 to 500 μm [31].

The complex filling field can affect the crystallization phase, which can be seen from the huge difference of the proportion of the skin and core layer in the “skin–core” structure for semi-crystalline materials [26]. For semi-crystalline materials, physical properties, such as mechanical, optical, electrical, and chemical properties, mainly depend on the crystalline morphology of the materials [27]. By analyzing the crystallinity effect of molded samples with different sizes along the flow direction, Liu et al. [28] studied the difference between macroscopic and microscopic morphology of isotactic polypropylene (iPP). The results show that there is also a “skin–core” structure with through-thickness morphology in micro-parts, which is similar to macro-parts, as shown in Figure 2a. At the same time, as far as the orientation area including surface layer and shear layer is concerned, the micro-part (90%) is much larger than the macro-part (15%) [29]. Zhang et al. [30] also found that the volume ratio of the skin layer increased from 10% to 67% as the part thickness decreased from 500 to 100 μm, as shown in Figure 2b. In addition, it was found that the Young’s modulus, fracture strain, and yield stress usually increase with an increase of the skin ratio [31]. The different molding conditions of micro/nano scale and macro scale determine the physical and chemical properties of injection parts by affecting the crystal morphology and proportion.

**Figure 3:**
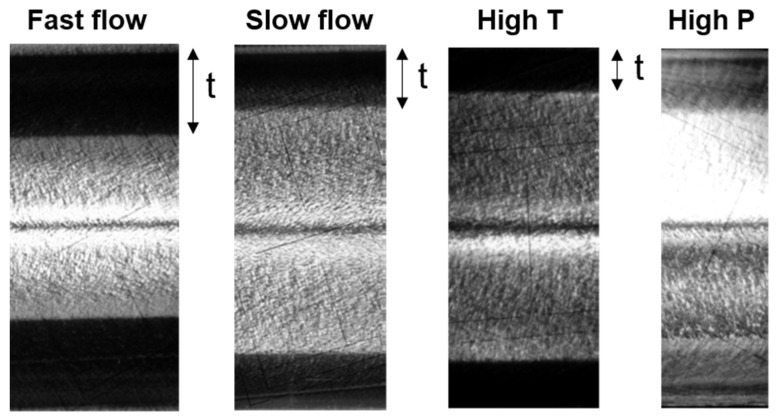
“Skin–core” structures at different conditions; T represents temperature, P represents pressure, t represents the thickness of skin layers [27].

The problems that micro-injection-molding technology is facing also have been discussed in some related literature [1,32,33]. Generally, to achieve the better molding filling process or morphology distribution, it is necessary to apply a faster injection speed [34,35], higher mold temperature [36], higher injection pressure [24], or surface coating technology [37] during the injection molding, as shown in Figure 3. In the injection-molding process, the influence of operating conditions on the internal morphology distribution of semi-crystalline polymer mold parts has been well summarized by Pantani et al. [27]. However, the introduction of the ultrasonic energy field lowers the level of these parameters. The general choice of researchers is to improve the parameter levels of certain factors in an attempt to associate the factor with an easily available result. Most of the research work in the past focused on the effect of crystallinity on viscosity, especially the description of the flow-induced crystallization phenomenon. However, it is still a question of which variables or their combinations are the most suitable for describing the evolution of crystal morphology.

### 2.2. Principles and Methods

#### 2.2.1. Process Principle

Due to the filling problem that reduced scale brings, the ultrasonic vibration was introduced to the MIM system to improve the filling capability by improving the fluidity of polymer melt. Since the application of ultrasound technology in polymer micro-injection molding has not been systematically reviewed, the names of similar technologies have not yet been unified: ultrasonic injection molding (UIM) [18], ultrasonic vibration micro-injection mold (UVMIM) technology [38], ultrasonic-assisted injection molding (UAIM) [39], but the common point of this technology is that the ultrasonic action is aimed at the polymer melt, improving its fluidity and achieving high replication and quality requirement of parts. Polymer plasticization, metering, injection, and other processes rely on existing micro-injection-molding machines (ARBURG [31,40], FANUC [14,41,42], BOY [43], Wittmann-Battenfeld [44]). Considering the difference between micro-injection molding and injection molding and the auxiliary effect of ultrasound, this kind of technology can be classified as ultrasound-assisted micro-injection molding (UAMIM). Although the internal morphology of micron-sized parts made of ordinary pure polymer materials has been widely studied and well understood, few papers have studied the morphology evolution of molded parts under the action of ultrasonic field: this problem has not been solved.

**Figure 4:**
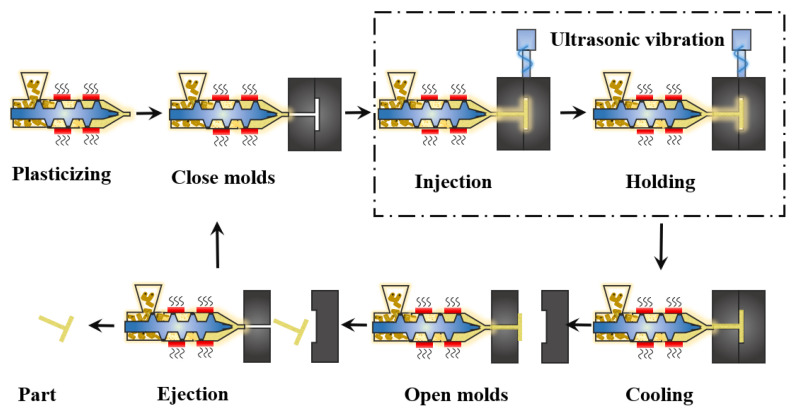
Schematic diagram of UAMIM [45].

UAMIM is a manufacturing process in which polymer pellets are plasticized by the conventional heating and screw shear heating in an MIM machine, and then, the polymer melt is injected into the mold cavity with the help of ultrasonic vibration, which is accompanied by energy exchange. Figure 4 shows the schematic diagram of UAMIM. The very high frequency of ultrasonic waves makes it possible to ensure the propagation direction in a very narrow space, which is not available for audible sound [45]. Ultrasonic energy exerts great acceleration on medium particles during transmission, which significantly affects the energy of medium materials [18]. Therefore, when the ultrasonic waves are applied to polymer melt, the fluidity of the polymer melt and molding quality of micro-parts are enhanced by the propagation of ultrasonic vibration energy in the polymer [45].

#### 2.2.2. Configuration Design

**Figure 5:**
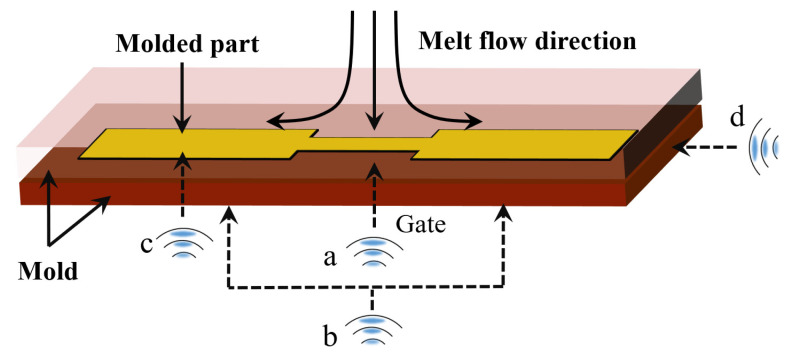
Different setting points of ultrasonic vibration.

As the auxiliary force field of ultrasound, the most important point is to transmit the vibration energy to the polymer melt during the filling process as much as possible. There are multiple options for the choice of the ultrasonic vibration point for this. Table 1 lists some common ultrasonic vibration point arrangements. Figure 5 shows different setting points of ultrasonic vibration. In terms of contact with the melt, it can be divided into direct ultrasonic vibration and indirect ultrasonic vibration. In indirect ultrasonic vibration, the ultrasonic sonotrode is not in direct contact with the polymer melt, and the ultrasonic transmission is indirectly transmitted to the polymer melt in the micro-cavity through the mold itself. As a result, a high-power ultrasonic energy field is applied to the mold, and high energy consumption and low effective utilization of ultrasonic energy have become the biggest challenges for the popularization of this process. In direct ultrasonic vibration (point c in Figure 5), the ultrasonic waves directly contact the polymer melt and apply high frequency vibration. This process requires a special ultrasonic sonotrode as a part of the cavity, which may cause problems such as its wear of and leakage of the rubber material.

When the molten polymer flows through the ultrasonic vibration application area, it will follow the vibration and absorb ultrasonic energy. However, when the melt flow direction is perpendicular to the ultrasonic vibration direction, there will be slight flow resistance in the melt, as shown by point b in Figure 5. If the vibration point is at the entrance (point a), the resistance will fill the melt into the cavity from the vibration point; therefore, the weight of molded parts will increase [18], and the vibration part of the ultrasonic waves will produce a sinking mark. If the vibration point is at the mold cavity (point b) or in direct contact with the melt (point a), the shear flow resistance will be weakened by ultrasonic vibration, the melt flow length will increase [39], and the filling percentage will also be improved [18,46]. The way the vibration direction is parallel to the melt flow direction (point d) can force more of the melt to maintain a straight flow over a longer distance [43]. To change the direction of ultrasonic action, the directional converter of ultrasonic waves is often used by researchers [39,47]. Hence, the vibration point should be set according to the structural characteristics of the injection part. Finally, since the flow viscosity of the polymer melt is affected by the application direction and transmission mode of the ultrasonic energy field, the vibration point should be set according to the structural characteristics of the injection part. However, the instructive suggestions for setting vibration points still need to be further studied and standardized more systematically.

### 2.3. Engineering Characteristics

#### 2.3.1. Improved Flow Properties

**Figure 6:**
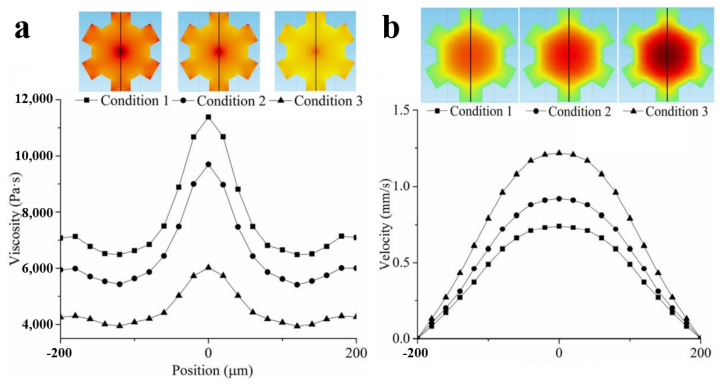
(**a**) Viscosity and (**b**) velocity distribution of polymer melt along the vertical centerline of the cross-section under different conditions [59]; Condition 1—without ultrasonic; Condition 2—with the flow frontier vibration; Condition 3—with the flow frontier vibration and changed rheological equation.

For UAMIM technology, the plasticization is completed by a traditional micro-injection-molding machine, and the ultrasonic force field is applied to the subsequent injection filling process. Numerous experiments and simulations have confirmed the positive effect of ultrasound on the filling rate [52,53]. The introduction of an ultrasonic field can slow down the cooling rate of molten polymer, prevent adhesion, reduce the thickness of the condensate layer, and prevent excessive shear forces and flow resistance, which leads to longer flow length and molding weight [39,45,54,55]. The decrease of flow resistance makes injection pressure reach the end of the cavity successfully, thus reducing the pressure loss between the proximal and distal ends of the gate. The pressure loss before and after the vibration area was measured by Yang et al., and it was found that the pressure loss is more obvious in thinner parts, and the pressure loss value of UAMIM is close to 27% compared with MIM [31]. Later research showed that a short shot can also be reduced with an increased ultrasound power, and a higher mold temperature is good for slowing the cooling rate and reducing the shear stress [46]. More intuitively, the filling process can be visualized with a high-speed camera [56,57,58]. In order to understand the flow behavior of molten polymer in the ultrasonic vibration injection-molding process, Jiang et al. [43] developed a new visualization device for analyzing the filling velocity field in the injection-molding process. The results showed that when the ultrasonic power is 200 W, the filling front velocity can be increased by 27% by ultrasonic vibration. In addition, during ultrasonic vibration, the velocity gradient of the melt decreases, and the velocity distribution became more uniform. The simulation results of Gao et al. [59] show that the ultrasonic vibration can obtain higher speed, lower viscosity, a more uniform viscosity field as shown in Figure 6, and better mold-filling performance, which makes the mold-filling quality of micro-plastic parts better, which is consistent with the experimental result before Qiu [45].

#### 2.3.2. Improved Replication Fidelity

The replication degree of molded plastic parts is an important criterion for judging the molding quality. The improvement of the melt quality under the effect of ultrasound vibration has a positive effect on the improvement of the replication of the molded part. Sato et al. [18,48] studied the effect of ultrasonic vibration on the quality of plastic parts. The results show that the replication performance of lenses is improved from 84% to 95% and the surface accuracy is improved by nearly 25%. The same result was obtained in a forming experiment involving a linear microchannel product. Qiu et al. [45] applied ultrasonic vibration to the mold-filling process of Fresnel lenses. The results were that the filling area of the plastic parts was improved by about 6.91% and symmetric deviation was improved 15.62% on average because of ultrasonic vibration. Lateral experiment and simulation proved it again [49,59]. The improvement of the mass or the filling length of molded parts is related to the temperature change under the action of ultrasonic vibration field. The temperature increases of the oscillation part of the ultrasonic wave (∆*T*) is shown as Equation (1).

(1) $$\Delta T=It\left(1-e^{-2\alpha X}\right)/\left(X\rho H\right)$$

where *I* denotes the sound intensity, *t* is the oscillation time, α is the coefficient of ultrasonic energy absorption, *X* is the distance, ρ is the density, and *H* is the heat capacity. 

**Figure 7:**
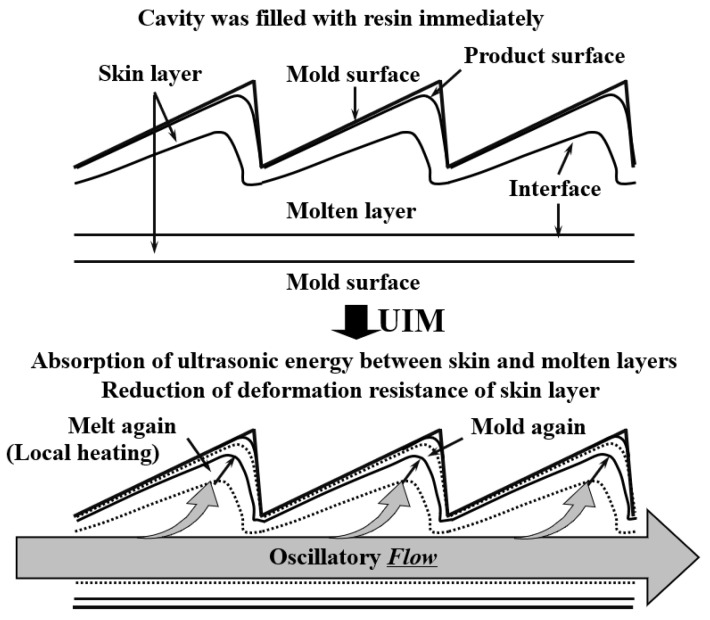
Improving surface replication mechanism by ultrasonic vibration [48].

Due to the absorption of ultrasonic energy, local heating occurs in the resin, which leads to the formation of oscillating flow in the packing and holding stages. The deformation resistance of the skin layer is reduced by the local heating occurring between the molten and skin layer. Therefore, as shown in Figure 7, surface replication, which mainly occurs in the packing and holding stages, is enhanced in the UAMIM process. Therefore, when producing micro-parts, especially with a functional surface microstructure, such as a microlens array, it is a feasible choice to apply an ultrasonic energy field to enhance the replication ability.

#### 2.3.3. Reduced Energy Consumption

In UAMIM technology, the enhancement of fluidity and the improvement of replication do not mean higher parameter settings. On the contrary, the auxiliary effect of ultrasound can reduce the parameter level required for material molding.

**Figure 8:**
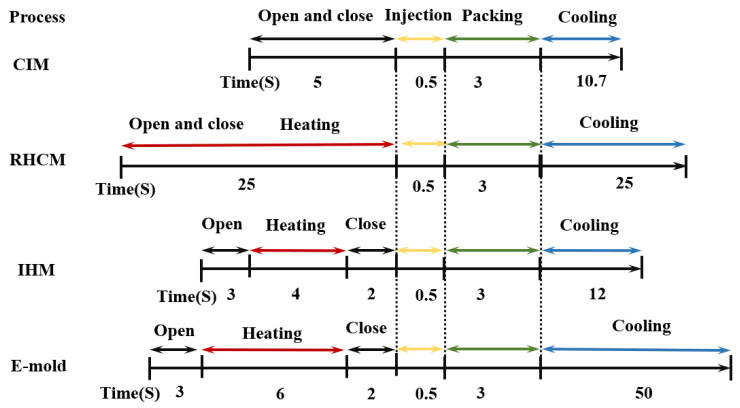
Time and production sequence required for each production cycle of different processes [62].

To achieve a molded part of higher quality, other assistive variotherm molding technology was used, including Rapid Heat Cycle Molding (RHCM) [60], Induction Heating Molding (IHM), and Electricity Heating Mold (E-mold). However, a higher process parameter setting, higher energy demand, and longer time are the cost. Chen et al. [61] systematic studied the electromagnetic induction heating system with a power of 30 kW to heat the mold plate. On the premise of low cost and practicability, Chang et al. [62] designed and investigated an infrared rapid surface heating system, which was used in the injection-molding process. In this system, the surface heat of a mold insert is provided by four 1 kW infrared halogen lamps as radiation sources. The former is almost 15 times the power of ultrasound and the latter is two times (the maximum power applied in UAMIM in the experiment is 2 kW, and even it is not more than 1 kW in practical applications). Using this kind of external heating technology, time to heat and cool was increased subsequently, increasing the whole cycle time. Figure 8 shows the time sequence of each cycle of the different processes. 

Since ultrasonic energy dissipates energy more quickly than heat conduction, the cycle time can be reduced. Liu et al. [38] compared two technologies in molding a high aspect ratio microstructured surface. One is micro-injection compression molding equipped with RHCM and vacuum mold venting (VMV), and another is micro-injection molding with ultrasonic technology (UAMIM). The results showed that all the process parameters in the latter one are lower, while a higher molding height was obtained, as shown in Table 2. In UAMIM, ultrasonic vibration only works on the microchannel, and the heat storage is little. Therefore, the cooling time required is only 10 s. Excluding injection time and demolding time, the production cycle in ultrasonic vibration micro-injection molding (*μ*UVIM i.e., UAMIM) is only 10 + 1.86 = 11.86 s, while micro-injection compression molding (*μ*ICM) needs to consider vacuuming time, with the production cycle of 20 + 25 = 45 s. Therefore, using ultrasonic technology, the production efficiency has increased nearly three times. Additionally, there is no need to equip RHCM and VMV in the UAMIM process; hence, the manufacturing cost of the mold is relatively low. Ultrasound in previous rheological tests has also shown that it can reduce the mold temperature parameter settings during polymer processing as well as the obvious viscosity drop and pressure reduction, which means that the molding cost is smaller. This has been confirmed in the injection experiments of Yang et al. [39,46], Jiang et al. [43], and Masato et al. [44] Although the ultrasonic energy field can reduce the energy consumption of molding, it is difficult to quantitatively judge its advantages only by the reduced parameter level. The quantitative work of related indicators such as power consumption and its reduction rate still need to be clarified.

In the process of injection molding, low-frequency vibration is also used to improve the mechanical properties of high-density polyethylene (HDPE) and iPP, such as reducing warpage [63,64], reducing residual stress [39], and improving welding strength [40,51,65,66]. This technology can also be used in the ejection stage and reduce the friction force during ejection [44]. Table 3 provides some successful cases in the ultrasound-assisted microinjection-molding process and their main characteristics.

### 2.4. Theoretical Interpretations

As an important link in the so-called “chain of knowledge” reaching from the production of polymers to their end-use properties, rheology plays an important role in polymer research [67], which seems to be more important in the micro/nano scale during MIM [68]. The improvement of the melt properties mentioned above under the ultrasonic field is ultimately the improvement of the polymer rheological properties. It is worth mentioning that when the channel size is less than 10 μm, the micro viscosity and wall slip play a vital role [69].

#### 2.4.1. Viscosity in Liquid

The index that measures the deformation resistance of fluid at a given rate is called the viscosity of fluid. “The viscosity of syrup is higher than that of water” is a common statement, in which the concept of viscosity corresponds to the informal concept of “thickness” in terms of liquid [70]. Shear thinning is the most common type of non-Newtonian behavior of fluids; as a kind of non-Newtonian behavior, the viscosity of polymer decreases with increasing shear rate or shear stress. The time-independent relationship between shear rate ($\dot{\gamma }$) and shear stress (*τ*) of non-Newtonian fluids can be described by the general Equation (2)

(2) $$\dot{\gamma }=f\left(\tau \right).$$

The behavior of fluids in the shear-thinning regime can be described with the power-law equation of Oswald and de Waele:

(3) $$\tau =K\left(T\right){\left[\frac{dy}{dt}\right]}^{n}=K\left(T\right)\dot{\gamma }.$$

This equation may be written in logarithmic form:

(4) $$\log\left(\tau \right)=\log\left(K\right)+n\log\left(\dot{\gamma }\right).$$

A log–log plot of shear stress (*τ*) versus shear strain (*dy/dt*) should yield a straight line if the polymer solution or melt behaves as a pseudoplastic liquid. The apparent viscosity is defined by the following Equation (5).

(5) $$\eta =\frac{\tau }{\dot{\gamma }}$$

A second power-law equation for the apparent viscosity is obtained by combining this expression with the Oswald Equation (6):

(6) $$\eta =K\left(T\right){\dot{\gamma }}^{n-1}$$

where *K* is the flow consistency index, *n* is the flow behavior index, for shear thinning or pseudoplastic flow, and *n* < 1.

As far as viscosity measurement is concerned, capillary viscometer and slit viscometer are two commonly used technologies for measuring melt viscosity [71]. By using a slit/capillary die embedded in a nozzle or mold, some work has been done to test the rheological properties of material with an injection-molding machine or an extruder [72,73,74,75].

The rheological test under ultrasonic field is more focused on the field of extrusion molding, but its influence mechanism is also of great significance for the application of ultrasound in the injection-molding process. The mechanism and influence of ultrasonic energy field on viscosity in the process of micro-injection molding need to be further studied. 

**Figure 9:**
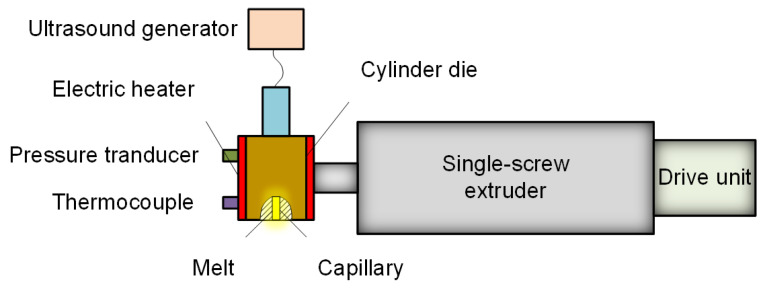
Specially designed ultrasonic oscillation extrusion system.

**Figure 10:**
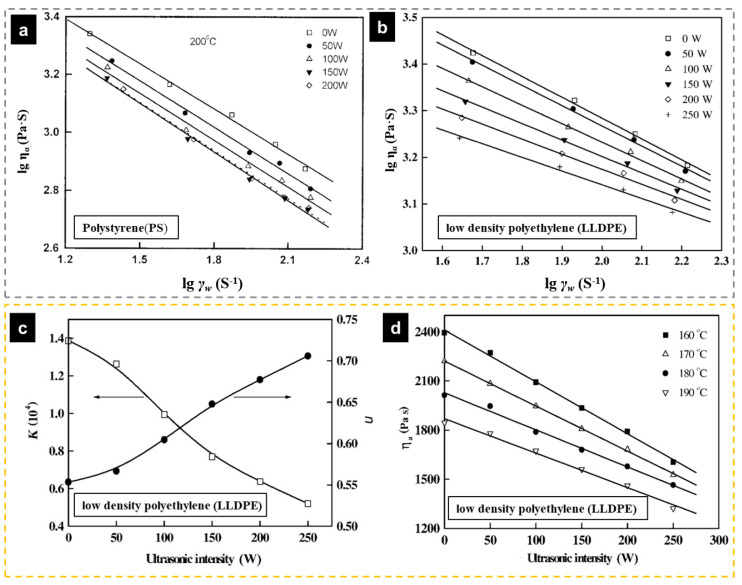
(**a**) Apparent flow curves of PS for different ultrasound intensities at 200 °C; (**b**) Apparent flow curves of LLDPE for different ultrasound intensities at 180 °C; (**c**) Correlation between K and n values of LLDPE under ultrasonic intensity at 180 °C; (**d**) Apparent viscosity of LLDPE vs. ultrasonic intensity at different temperatures ($\gamma_{\omega }$ = 80 s^−1^) [77].

Under different ultrasonic power levels, a linear relationship between log$\;\eta_{a}$ (logarithmic viscosity) and log$\;\gamma_{w}$ (logarithmic shear rate) was detected in the ultrasonic-assisted extrusion process, which obeys the equation. Based on the power-law model, Guo et al. have done a lot of work about the effect of ultrasound on rheology improvement using the specially designed ultrasonic oscillation extrusion system, as shown in Figure 9. The results showed that when ultrasonic vibration is introduced into PS melt, *n* increases with the increase of ultrasonic intensity, which indicates that ultrasonic vibration reduces shear sensitivity. With the increase of ultrasonic intensity, the consistency index of polystyrene decreases, indicating that the melt viscosity of polystyrene decreases under the action of ultrasonic oscillations [76]. The same trend is obtained in LLDPE [77], as shown in Figure 10, and HDPE [78]. The most intuitive conclusion is that at different temperatures, the apparent viscosity of the melt decreases with increasing ultrasonic density. In other words, when the apparent viscosity is maintained at the same level, ultrasonic vibration will lower the processing temperature of LLDPE. Later studies on other polymer materials show the similar trend via ultrasonic processing. 

However, ultrasonic energy field can transform vibration energy into heat energy to increase the melt temperature. It is difficult to rule out the possibility that the decreased viscosity is caused by the increased melt temperature. The different application methods (direct or indirect) of the ultrasonic energy field, the setting of the vibration point (parallel or perpendicular), and the energy utilization efficiency are still unresolved.

The orientation of molecular chain flow is the main reason for the elasticity and non-Newtonian property of polymer melts. It is said that entanglement of molecular chains decreases the possibility of molecular chain orientation and slows down relaxation, while ultrasonic vibration can shorten relaxation time, obviously [79]. Chen et al. [79] found that the apparent relaxation time dropped sharply by increasing the ultrasonic intensity from 0 to 50 W. Molecular chain disentanglement, which orients molecules, can be attributed to the strong vibration, crushing, and cavitation of power ultrasound, which is helpful to excite and activate molecular chains [80,81]. In fact, at a sufficiently high shear rate, highly anisotropic polymer chains can be disentangled and aligned along the shear direction [82]. Therefore, the viscosity of polymer melt can be reduced by fewer molecule/particle interactions and larger free space [83]. Additionally, the influence of power ultrasound on the physical and chemical properties of polymer melts is also one of the important research contents. The research shows that under most research intensity, the influence of chemical effect on the apparent viscosity of polypropylene accounts for 35–40% of the total ultrasonic effect [84]. 

The above reports sufficiently explain why ultrasonic vibration can improve the quality of molded parts, increase the replication fidelity, and even reduce the viscosity. Interpretation at the molecular level is conducive to the design and optimization of process parameters. At the same time, this may be the most significant advantage of the UAMIM process.

#### 2.4.2. Wall Slip in Cavity

Generally, viscous fluid means adhering to the boundary and reaching the boundary velocity in the process of flow. However, the so-called “wall slip” here means that there is a relative velocity on the contact line between the fluid and the solid boundary during the flow process [85].

**Figure 11:**
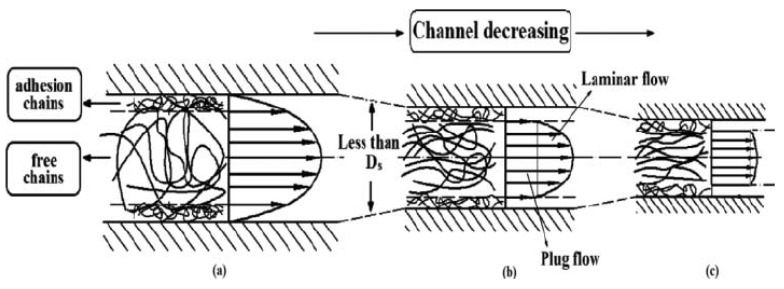
Polymer flows through microchannel with size less than D_s_ (0.2–0.5 mm); (**a**) laminar flow; (**b**) plug flow and laminar flow; (**c**) Newtonian fluid [93].

There are two slip regions in the flow of molten polymer. When the wall shear stress exceeds a critical value, molten polymers slip macroscopically over wall surfaces, which is known as weak slip or adhesion-detachment wall slip [86,87,88,89]. Then, for the characteristics of linear polymers, especially those with relatively narrow molecular weight distribution [90], when a second critical value is exceeded, another slip will occur, which is called strong slip or entanglement-disentanglement wall slip [42,90,91,92]. Deng et al. [93] found that with the decrease of channel size (the increase of shear stress), the destruction speed of the entanglement points was faster than the reconstruction speed, as shown in Figure 11. When the diameter of the channel decreases further and the shear rate increases to a certain value, there is not enough time to reconstruct the entanglement points. Hence, the free chains can be easily oriented along the velocity field, but those in the attachment area remain entangled and attached to the wall.

Slip plays an important role in correctly determining the rheological properties of polymers [94], which can correct the slip effect data and explain the reasons for the mismatch of rheological data obtained from various rheometers with different geometries [95]. In micro-injection molding, the no-slip boundary condition is not valid due to the high velocity, high pressure, and micro-scale flow conditions; hence, the melt flow will exhibit uneven and complex changes in the micro-cavity. Especially in the micro-sized cavity, because the shear stress is greater than that of the conventional cavity, the occurrence rate of wall slip is higher in the filling process [96,97]. 

The molding quality of micro-polymer parts is largely controlled by the melt flow field in the micro-cavity, in which the influence of wall slip is complex and cannot be ignored [98]. The influence includes significantly reducing the wall shear stress and the melt apparent viscosity, reducing the velocity gradient, improving the uniformity of flow rate distribution and viscosity distribution, and thus promoting the mold filling in micro-cavity, or further transforming the flow field into a piston flow, which is good for filling [99,100]. 

**Figure 12:**
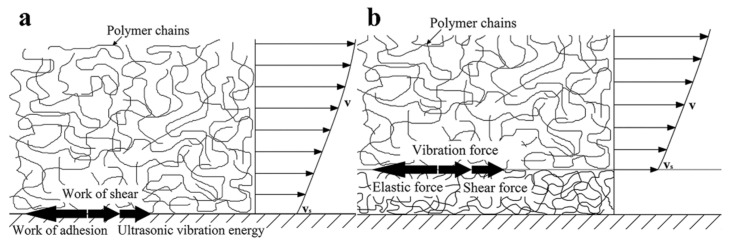
The schematic diagram of the weak slip (**a**) and strong slip (**b**) under ultrasonic vibration [42].

Although UAMIM has been proved the ability to improve the rheological properties of polymer melt and promote melt flow, there are few related research studies on the mechanism of wall slip under an ultrasound field. Gao et al. [42] established both adhesion–detachment and entanglement–disentanglement wall slip models by combing the effect of ultrasonic vibration. The results measured in the home-made equipment show that both weak and strong wall slip of melt in a micro-cavity can be enhanced by ultrasonic vibration, which agrees with the built theoretical models, as shown in Figure 12. Ultrasonic vibration can reduce the apparent viscosity of polymer melt, release shear stress, and improve the filling ability of melt in the micro-cavity, thus improving the molding quality of micro-polymer parts [45]. With the introduction of a new physical field, it is necessary to customize viscosity testing equipment for polymer rheology, and there are few reports in this field. At the same time, the test standards and test conditions of viscosity and wall slip should not be excluded from the discussion.

## 3. Ultrasonic Plasticization Micro-Injection Molding

### 3.1. Size Effect in MIM

The size effect is mainly reflected in the situation in which the volume and the injection volume of the final molded part are seriously mismatched. Almost all micro-injection-molding machines cannot avoid material waste caused by miniaturization.

Three-section screws are used to plasticize polymers commonly in the processing. The screw diameter is limited due to the size of standard granules and large shear force in processing, making the smallest diameter about 14 mm, and the screw moves just 1 mm. About 0.185 g of plastic material are injected [19], while a part can only be 0.024 g or less, which means 0.185–0.024 g of material is wasted. In the production of medical devices, it is extremely important to avoid wasting raw materials that directly affect the price of parts, because the cost per gram of bio-absorbable polymers may be 5–10 dollars [21].

In order to achieve a small injection volume (<1 mg), IKV (Institute für KunststoffVerarbeitung, Aachen, Germany) has prepared a small desktop-level micro-injection-molding machine that is only the size of a shoe box, in which 2 mm injection plungers, 5 mm metering plungers, TOOLVAC technology^TM^-have been used [101]. However, plasticization time has become a large proportion of the entire molding cycle due to the electric heating plasticization; hence, ultrasonic plasticization of trace polymers was investigated in order to improve the efficiency by Michaeli et al. [19], and the molding process based on this kind of plasticization method is called UPMIM. It is reported that the injection volume can be distributed between 5 and 300 mg. The optimization of the structure, although fully electrified to some extent, alleviates the problems caused by miniaturization, but it is still far from enough. UPMIM technology with a simple plasticization and molding principle has generally demonstrated its characteristics and advantages since its introduction in 2002. Obviously, this technology provides a new choice for the injection molding of small batch micro-parts.

### 3.2. Principles and Methods

#### 3.2.1. Process Principle

**Figure 13:**
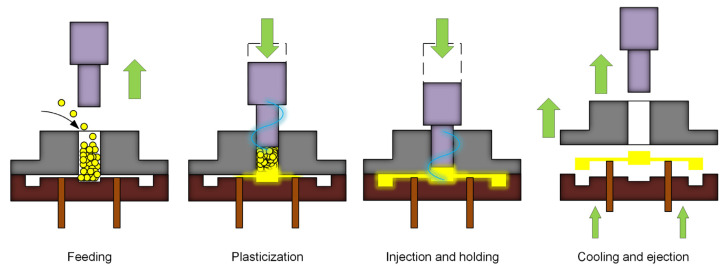
Schematic representation of the UPMIM process.

Similar to conventional micro-injection molding, UPMIM has the same molding stage: feeding, plasticization, injection and holding, and cooling and ejection. The schematic of the UPMIM process is shown in Figure 13. Since UPMIM is designed to plasticize a small amount of polymer in a single shot, only one molding cycle of raw materials is needed at the feeding stage. During feeding, the sonotrode is displaced upward to leave the feeding space. Then, it is displaced downward to provide sufficient pre-compression to plasticize the polymer particles at high frequency. During the injection and holding stage, with the downward movement of the sonotrode, the filling of the polymer melt is completed. After the part is cooled down, the rod ejects the part, and a molding cycle is completed, and the second molding can be performed by feeding. There are various names for this process, such as ultrasonic molding [102,103], ultrasonic injection molding [21], ultrasonic micro molding [104], ultrasound injection molding [105], and ultrasonic micro-injection molding [106]. In order to avoid confusion with ultrasound-assisted micro-injection molding (UAMIM) and ultrasonic compression molding (UCM), ultrasonic plasticization micro-injection molding (UPMIM) is named after its most important process feature, that is, power ultrasound is the only energy source for plasticizing polymers.

In terms of molding process, only a small amount of raw materials are plasticized and injected in a single cycle of UPMIM. Therefore, it seems that reducing cycle time and material waste, especially medical materials, is the main advantages of UPMIM. Furthermore, the process can save production costs when producing small batches, and it is especially suitable for the initial stage of product development. However, the micro-injection-molding process still has irreplaceable advantages in mass production and automated production.

#### 3.2.2. Configuration Development

The UPMIM equipment currently used for molding can be divided into the following: (1) an independently developed ultrasonic plasticizing experimental platform, including an upgrade by an ultrasonic welding machine [19,102,107,108,109], completely home-made equipment [20,110,111]; (2) commercial equipment: Sonorus 1 G has a maximum single injection weight of 2 g, but for the needs of the industry, this machine can accommodate a slightly larger shot size [106,112,113,114,115]. Subsequently, the second version of the equipment (the Sonorus S210 machine) came out in 2016, with a maximum injection weight of 5 g. As for the driving method, the ultrasonic plasticizing experimental device was driven by the previously unstable air pressure [108,112] and replaced with a servo drive with precise displacement and pressure control [20].

**Figure 14:**
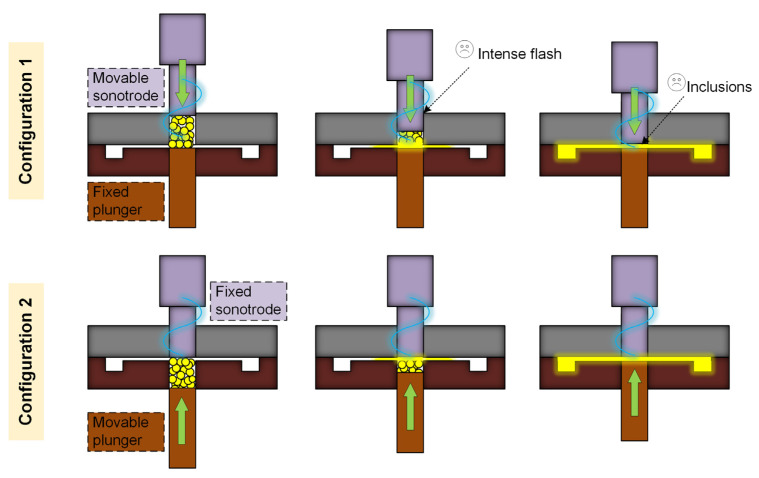
Schematic representation of “injection while plasticizing” processing mode in UPMIM.

In terms of UPMIM processing modes, they can be divided into two categories. The first type is “injection while plasticizing”. Another type is “injection after plasticization”. For the former, the plasticizing cavity and the cavity are connected. Ultrasonic plasticization is accompanied by injection filling of the melt. At present, almost all related equipment operations adopt “injection while plasticizing” mode. Under this mode, the structure of the equipment can be divided into two configurations, as shown in Figure 14. In Configuration 1, the lower plunger is fixed during the molding process, and the ultrasonic sonotrode performs ultrasonic vibration while completing the injection-filling operation. In Configuration 2, the ultrasonic sonotrode has no displacement during the molding process, and it is only responsible for ultrasonically plasticizing the polymer. The injection and filling behavior are completed from the bottom to the plunger. The most intuitive performance of the two frame structures is that the end face of the sonotrode of the latter is always flush with the surface of the cavity, and the position of the sonotrode of the former has been dynamically changed, which is one of the reasons for the surface wear of the sonotrode. The polymer on the surface of the sonotrode in Configuration 1 is plasticized first, and the energy is transferred from the top to the bottom. During the injection filling, the unplasticized impurities easily enter the cavity, and Configuration 2 can reduce the wear and uneven filling to a certain extent. Reports in recent years have shown that Configuration 1 is being replaced by Configuration 2 with obvious advantages. However, no matter which configuration it is, more profound basic theoretical research is needed. Only in this way can the process stability be improved. On the other hand, the “injection after plasticization” mode is superior in process stability. Compared with the former, the latter not only uses an ultrasonic energy field to plasticize raw materials but also includes metering and injection devices. In fact, almost no current equipment adopts this mode. However, well-designed equipment according to this model still has a chance to be one of the potential development directions.

### 3.3. Engineering Characteristics

#### 3.3.1. Increased Material Utilization

**Figure 15:**
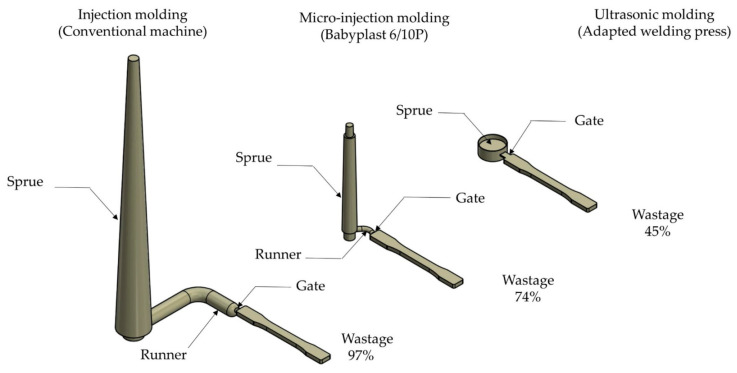
Qualitative comparison among different feeding systems [21].

There is still a runner and sprue in molded parts, which made it appear particularly large in micro molding. However, compared with traditional MIM, UPMIM can save 40% to 70% of the equivalent cold runner. The volume of the cylindrical gate depends on the diameter of the sonotrode and plunger, and the commonly used size is 8 mm [108,112] and 10 mm [20,111]. In the experiment of Sacristán et al. [112], polylactide (PLA) was used to mold eight test specimens of small dimensions, each weighting 10 mg, where a shot weight of 250 mg is required. Finally, nearly 70% of the loaded materials became waste (170 mg), which were wasted on the sprue and runners, saving 20% of the materials compared with the traditional injection-molding system [19]. In another UPMIM equipment, Grabalosa et al. [108] reduced the waste to 45% according to calculations of Heredia et al. [21]. Figure 15 shows the quantitative comparison of waste materials among the feeding subsystems in different micro-part production systems. For some high-performance polymer, such as polyetheretherketone (PEEK), the polymer itself is not only expensive but also requires processing at high temperatures and employing additional special equipment which, in turn, further increases the cost of production, which renders the PEEK injection micro-molding process uneconomic for low volume series and customized micro-parts [116]. Dorf et al. [117] analyzed the influence that the main process parameters have when processing the PEEK polymer via UPMIM successfully; the results demonstrated the fact that UPMIM technology is capable of producing parts with competitive properties. In terms of a material utilization ratio, the smaller the molded part, the more obvious the advantages of UPMIM, especially when the performance of some medical materials will be affected after secondary plasticization.

#### 3.3.2. Reduced Energy Consumption

**Figure 16:**
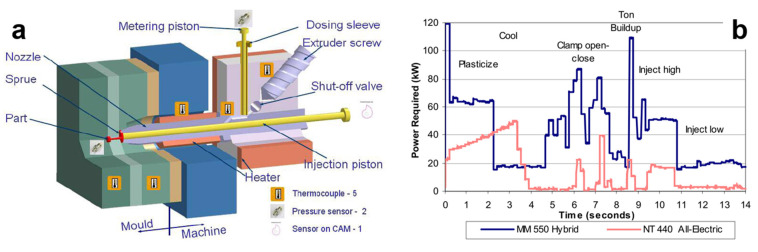
(**a**) Injection unit of the Battenfeld Microsystem 50 and the sensor installation positions [119]; (**b**) Comparison of energy consumption in injection-molding cycle between hybrid power (electric screw drive) and all-electric machine [118].

An all-electric molding machine (Battenfeld Microsystem 50) was designed for precise, small micro-parts by splitting plasticizing, dosing, and injecting unit. Its injection system consists of a screw plasticizing barrel, a plunger injection system, and a melt dosage control barrel, as shown in Figure 16a. 

In the MIM system, nearly 20% of the total energy in the injection process is used to heat the plasticizing unit [6]. According to the research of Spiering et al., about 40% of the total energy consumption is concentrated in the injection-molding process, including mold heating, mold moving, and melt injection [7]. In different types of energy generators such as electric, hydraulic, or hybrid, in fact, the most effective one should be the electric. The power requirements of hybrid and all-electric machines are shown in Figure 16b, both of which run the same components with a cycle time of 14 s. The results show that using all-electric hybrid technology can save substantial energy when the efficiency of the hydraulic machine is even lower than that of hybrid technology [118].

**Figure 17:**
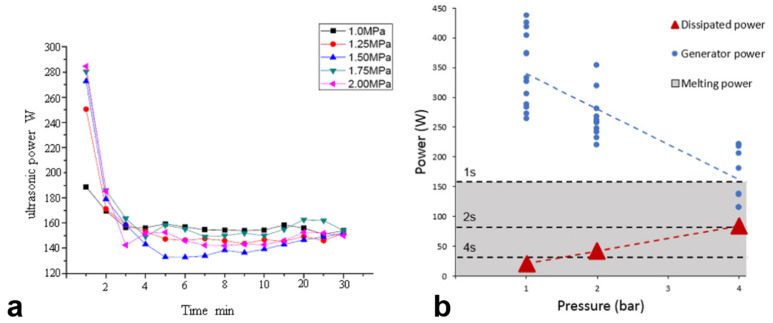
(**a**) Curve of ultrasonic power under different pressure [120]; (**b**) Comparison between generator, dissipated power, and required average power [108].

The plasticizing stage is the main energy-consuming stage in the UPMIM process. Compared with the relatively energy-saving full-electric micro-injection molding, the plasticizing stage also has greater energy savings. The plasticizing phase is performed by the sonotrode, and energy is provided by an ultrasonic power source. The rate power of most ultrasonic generators used now in UPMIM are 1 kW [112] and 1.5 kW [108]. In fact, under normal conditions, the required power is between 400 and 500 W. If the pressure of the end face of the sonotrode is overloaded, the power will exceed 1000 W, and the whole system will be unstable. Jiang et al. [120] showed that the ultrasonic power increases with the pressure at the first two minutes. The maximum plasticizing power does not exceed 290 W, as shown in Figure 17a. Grabalosa et al. [108] pointed out that the higher the pressure, the better the energy utilization ratio for melting the polymer (under the condition that the ultrasonic generator is not overloaded). Therefore, with the increase of pressure, the efficiency of the ultrasonic sonotrode plasticizing process will increase from about 10% to 50%. In any case, the maximum power of the generator can reach 150 W, as shown in Figure 17b.

On the other hand, molding a micro-part or structures requires less pressure and lower energy consumption in UPMIM. In traditional precision injection-molding machines, typically, 1600–3500 bar was used [1,108]. However, in the UPMIM process, these pressures drop to the range of 300 to 500 bar. For example, Sonorus 1 G is technically rated with a clamping force of 3 m.t., while 1.5 to 2.2 m.t. of clamping force is needed. In addition, the energy consumption of Sonorus 1 G is directly reduced by 85% to 90% compared with that of the standard injection-molding machine due to the elimination of the heater bands, hydraulic pumps, and motors, which are usually used to keep the clamp shut under high pressure. Grabalosa et al. [108] adopted an electro-pneumatic ultrasonic molding machine, which reduces the waste of micro-parts by nearly 10%. For the UPMIM equipment, all-electricalization is also a developmental trend.

Many reports have proved that the UPMIM process has lower energy consumption than the traditional injection process. However, in the process of mass production, the advantages of UPMIM are limited by its production capacity. It is difficult to explain how obvious this advantage is only through one or several cycles. The cost of manpower and time needs to be considered, and more research needs to be carried out.

#### 3.3.3. Reduced Residence Time

In the field of precision molding, material degradation may be the key issue that plastic parts manufacturers pay the most attention to. The residence time directly affects the degradation of materials, which is an inevitable problem in the configuration of a screw barrel and heater band in all traditional molding techniques.

**Figure 18:**
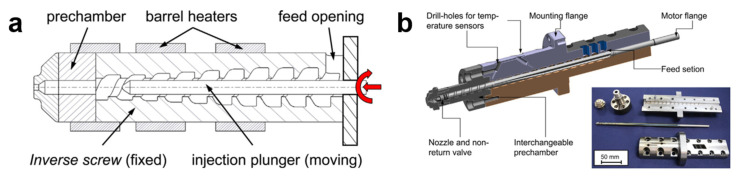
(**a**) Schematic diagram of inverse screw design; (**b**) Engineering case of injection-molding machine with inverse screw structure [5].

The innovative plasticizing unit, a so-called “inverse screw”, was designed by the German research institute IKV and Arburg company, which is applied in a new electric Allrounder 270A injection moulding machine, as shown in Figure 18 [5]. Therefore, the appearance of an inverse screw is an improvement to the processing of thermally sensitive and medically relevant materials such as polylactic acid.

Compared with traditional MIM, UPMIM has no plasticizing screw, screw barrel, heater band (not necessarily), etc. In terms of process, it only plasticizes the amount of material needed for each injection, and it melts in situ in the mold near the gate. This reduces the thermal history of plastics to several milliseconds, reduces the waste of raw materials, avoids degradation as much as possible, and even eliminates the need for material purging. UPMIM has been already proved able to mold parts made of PLA [112,121]. However, in the traditional micro-molding process, when the injection amount of a micro-part may only be 0.1 g, the machine has a 100 g capacity barrel, which indicates that the barrel must be emptied after nearly 1000 injections. Therefore, UPMIM provides a feasible solution for heat-sensitive and poorly stable materials. On the other hand, short residence time brings challenges to plasticization uniformity and stability, which is related to process maturity and stability. The synergistic principle and mechanism of some process parameters such as injection speed, ultrasonic amplitude, and power are conducive to enhancing process stability. 

#### 3.3.4. Improved Filling and Molding Ability

The fact that the viscosity of the polymer melt will decrease under the action of ultrasound has been confirmed by many scholars [76,84,122,123,124], which means that UPMIM can apply lower pressure at the same melting temperatures and can make the material flow into thinner, tinier geometries, which could not be filled previously. Although there are almost no reports about the UPMIM process used to produce industrial microfluidic devices, many researchers have explored its feasibility. In terms of filling capacity in UPMIM, Jiang et al. [125] used a spiral flow test based on an Archimedes spiral mold with microchannels (depth from 250 to 750 μm) to test the fluidity of molten polymer plasticized by ultrasonic vibration. The results show that with an increase of the ultrasonic amplitude, ultrasonic action time, plasticizing pressure, and mold temperature, the fluidity and filling length of polymer melt (PA66, PP, PMMA) can also be effectively increased. On the other hand, Ferrer et al. [126] proved the repeatability and reproducibility of processing a microchannel thin-walled plate in polystyrene polymer. The results show that the thickness deviation of the final part is less than 7%, and the reproduction depth of the microchannel is greater than the width, with average deviation of 4% and 11%, respectively. The authors also demonstrated the process feasibility to ratio parts of polylactide acid (PLA) by an ultrasonic molding process and discussed the feasibility of producing PLA products with a low aspect ratio [127]. Additionally, UPMIM was proved to mold another kind of micro-piece that requires high precision to replicate its details [112]. Among them, parts with small size details such as guitar strings, the width of which is 70 μm, can be molded well, which is difficult to produce by conventional micro-injection due to the high pressure requirements of machines [128].

The reduced melt viscosity and injection pressure seem to illustrate the improved injection-molding ability. However, due to the different plasticizing process, the UPMIM process can hardly control the melt temperature strictly at present. Hence, the same process parameter level cannot be strictly guaranteed, and the performance improvement caused by the miniaturization of equipment cannot be excluded. Furthermore, rapid thermal cycling technology can also improve the molding ability of micro-injection molding. In the end, UPMIM may have certain advantages in molding ability, but it is not enough to be a reason to completely replace MIM.

#### 3.3.5. Wear of Sonotrode

**Figure 19:**
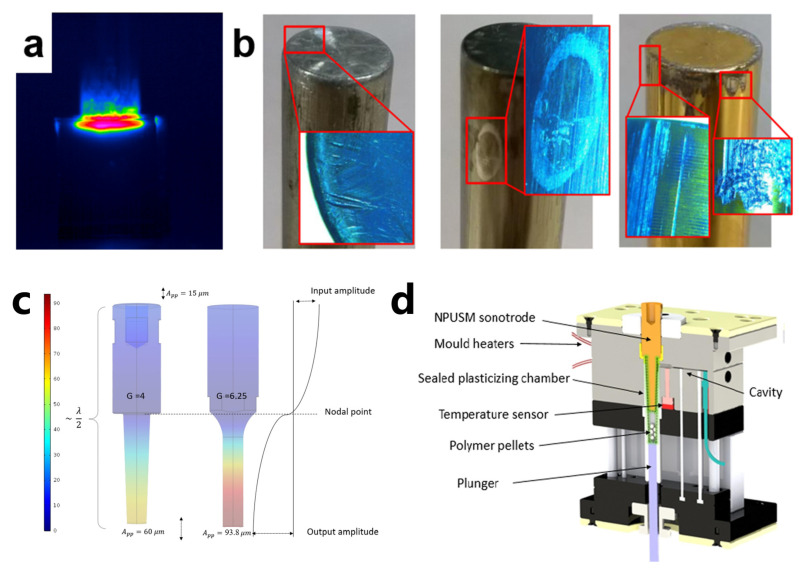
(**a**) Intense flash; (**b**) Wear observed in different sonotrodes used in UPMIM machines [102]; (**c**) A nodal point sonotrode (left) and a conventional one (right); (**d**) Nodal point ultrasonic micro-injection-molding configuration [130].

Regardless of whether it is Configuration 1 or Configuration 2, it is necessary to consider the axial positioning accuracy of the sonotrode and the plasticizing chamber as well as the tolerance (clearance fit) and other issues during assembly. Although the sonotrode is subject to longitudinal high-frequency vibration, lateral vibration will occur during the work process. Once the amplitude is greater than the amount of the fit gap, it will dynamically contact and wear with the side wall of the plasticizing cavity. This phenomenon is particularly obvious in Configuration 1. During the packing phase of Configuration 1, the sonotrode is displaced upward to leave space for particle feeding. After adding the required particles, it is downwardly displaced to pre-press the particles. Ultrasonic vibration is turned on for high-frequency vibration while accompanying with slight radial vibration. Pressing down the polymer particles will cause a sharp frictional heat generation in the plasticization chamber, as shown in Figure 19a, and the ultrasonic power will increase sharply, which will cause the frequency of the ultrasonic generator to exceed the vibration frequency range of the power supply. Wear also occurs after working, which can be classified into two types: longitudinal and lateral/diametrical wear, as shown in Figure 19b [102].

The mass variation at the tip of the sonotrode is reflected in the uneven wear, which will lead to the change of longitudinal vibration mode frequency. In addition, due to the inhomogeneity at the worn surface, stress concentration regions will appear, which will threaten the stability of the system and plasticizing process. In some experiments, the tip of the horn should be cleaned before action to ensure uniform contact with the sample [129].

According to the working principle and characteristics of ultrasonic probe, Janer et al. [130] solved the problems of sonotrode wear and flash by introducing the concept of a “nodal point” (position of the sonotrode for which its vibration is “zero”) and improving the structure and cooperation of ultrasonic sonotrodes, as shown in Figure 19c,d. At the same time, the quality of injection parts and the stability of quality have been improved to varying degrees. However, the utilization rate of materials decreased from 20% to 9% compared with that before upgrading. At present, it may be the best choice by sacrificing some other capabilities to extend the service life of the sonotrode.

#### 3.3.6. Instability of System

Configuration 1 and Configuration 2 have one thing in common: that is, a single direct gate is used to connect the mold cavity and plasticizing chamber. Communication between the plasticizing unit and the injection unit also directly causes the ultrasonic sonotrode to bear excess injection pressure during the plasticizing process, which in turn causes the process to be unstable. In the case of pressure applied in the experiment designed by Grabalosa et al. [108], when the injection pressure is higher than 2 bar (one bar measured by the manometer represents 311 N of force at the tip of the sonotrode), the forming rate of the part is 90%, while the injection pressure lower than 2 bar cannot even mold a complete part. However, the injection pressure higher than 5 bar will overload the ultrasonic equipment, because the excessive interaction force between the ultrasonic sonotrode and the material will make the vibration frequency exceed the rated value, thus interrupting the molding cycle. In reference to the molding force, it takes more than 300 N injection pressure to fully mold eight samples. However, the above situation does not mean that there is no upper limit on the applied pressure, because in some cases, the force of about 500–600 N means that the ultrasonic electrode will be overloaded (unable to vibrate) due to the high compression of PLA, that is, the system will be unstable [129].

**Figure 20:**
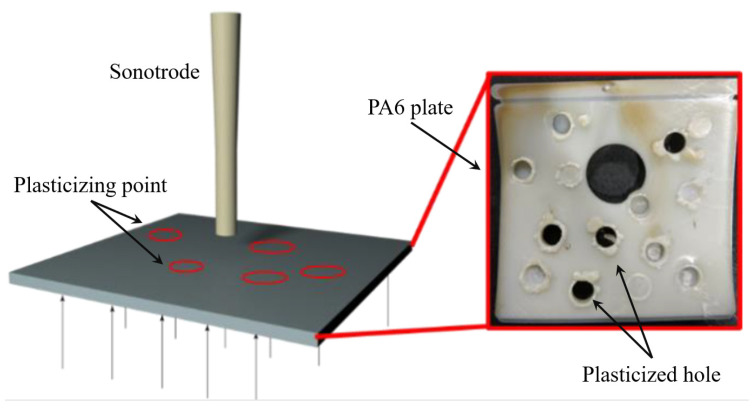
Ultrasonic plasticization test under low pressure.

In this regard, the author has done a test with a small lifting force (<300 N), loading on an ultrasonic sonotrode of 10 mm diameter and 40 kHz vibration frequency. A short glass fiber-reinforced PA6 plate can be easily plasticized and perforated, as shown in Figure 20. In other words, from the perspective of protecting the sonotrode and the power supply system, the working load of the ultrasonic sonotrode should be minimized while ensuring the plasticized state of the polymer. In the patent of Wu et al. [131], a solenoid valve was used to isolate the plasticizing cavity from the cavity. When the solenoid valve is open, the plasticizing cavity is separated from the cavity. After a certain period of ultrasonic action, the solenoid valve is closed, the plasticizing cavity and the cavity are connected, and the displacement of the sonotrode fills the polymer melt into the micro-cavity. Wu further imitated the three-stage injection-molding model of Microsystem 50 to form a three-stage ultrasonic plasticization molding process of plasticization, metering, and injection [132,133].

**Figure 21:**
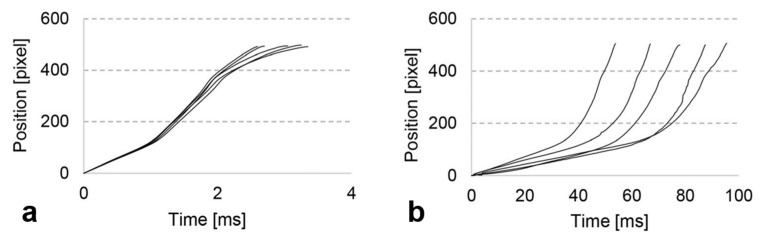
Flow front tracking for five repeated MIM (**a**) and UPMIM (**b**) experiments when the set flow rate is 5000 mm^3^/s [105].

The instability of the plasticizing stage will directly affect the subsequent injection filling. Through tracking of the flow front, Masato et al. [105] found that compared with the MIM process, the filling time of the UPMIM process is longer and more dispersed, as shown in Figure 21, which indicates that the process is less consistent and stable. The filling time in UPMIM is mainly controlled by the melting rate, which depends on the ultrasound vibration characteristics, while are difficult to control now. In the experiment of Dorf et al. [113], from the 196 combinations of parameters setting, only 47 sets allowed the cavity to be completely filled, which indicates the unstable parameter setting. 

**Figure 22:**
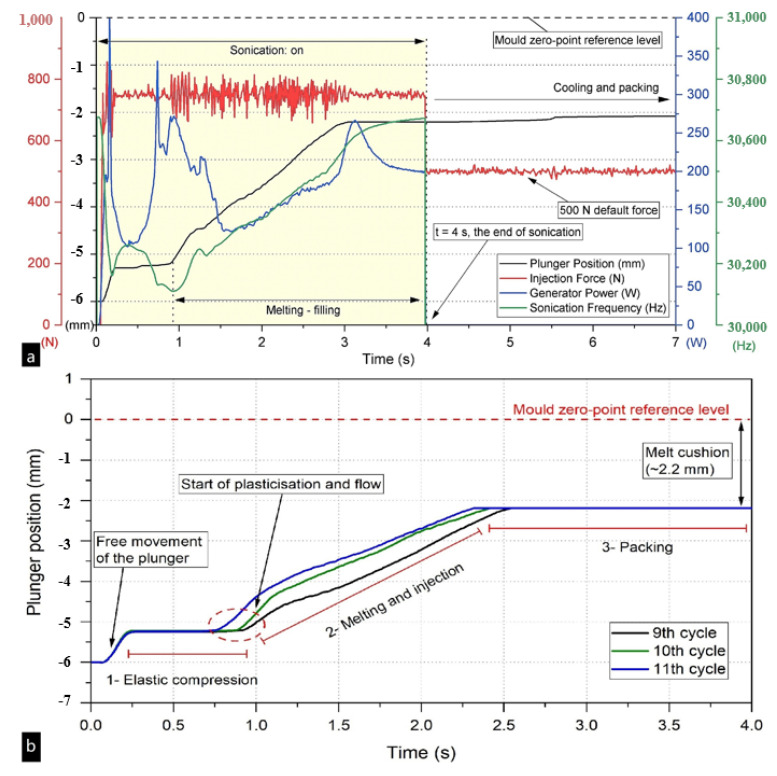
(**a**) Machine data captured during ultrasonic micro-molding of PP; (**b**) Plunger position recorded from three consecutive ultrasonic micro-molding cycles while the sonication was applied [134].

Gülçür et al. [134] evaluated the stability of UPMIM by an in-line monitoring method, which consists of a series of sensor technologies, including data recorded by the machine controller, a high-speed thermal camera, and a cavity pressure sensor. The results show that the data obtained from machine sensors is essential for understanding each stage of the ultrasonic micro-molding cycle, as shown in Figure 22. The plunger position is highly correlated to the characteristics of each molding stage, as shown in Figure 22a. Therefore, the different stages of the process can be easily tracked by analyzing the position of the plunger in detail. Figure 22b also shows that although each channel of machine data may change significantly with different cycles, the main features of the process plots are still displayed in each molding cycle. When the dynamic process environment of the UPMIM process is clearly described, the mechanical data captured in-line have a high accuracy. This technology can not only directly reflect the relationship between process parameters and the quality of molded parts but also meet the indicators of stabilizing part quality and tracing part defects. Wu et al. [135] proposed a new method to quantitatively characterize the efficiency of simultaneous plasticization and filling by redefining the injection rate as the mass flow during melt filling. The results show that without damaging the mechanical properties of the micro-molded samples, increasing the injection rate is beneficial to simultaneously increasing the efficiency of plasticization and filling. At the same time, Janer et al. [130] improved the quality of injection parts and the stability of quality to varying degrees by introducing the concept of a “nodal point” and improving the structure and cooperation of an ultrasonic sonotrode. In view of the results obtained in the above reports, deepening the understanding of UPMIM principles and forming mechanisms, especially the synergistic effects of various parameters, is the best choice to improve process stability, and there is still much work to be done.

#### 3.3.7. Uniformity of Molded Part

**Figure 23:**
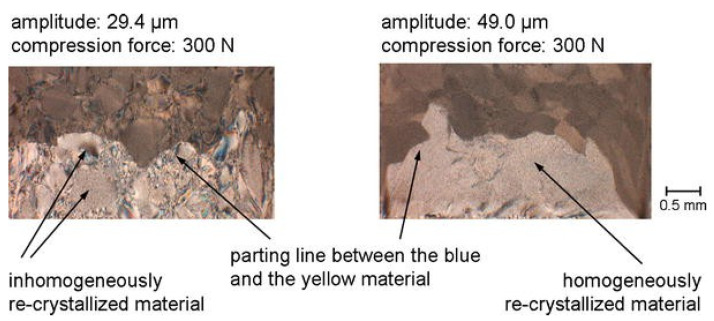
Effect of the mixing of colored powder (blue/yellow) during the plasticization: different microscopic appearance after changing the amplitude [107].

Whether it is MIM or UPMIM, the uniformity of polymer melt in the plasticizing process is an issue that attracts much attention. Molding quality and system stability are still the biggest challenges facing UPMIM. Unlike MIM processing, there is no screw in the UPMIM technology, meaning that it is necessary to test the mixing effects in UPMIM. Michaeli et al. [107] firstly tested the mixing effect of UPMIM; blue and yellow PP-powder was plasticized under ultrasonic vibration. Under the same pressure, a small amplitude (29.4 μm) will cause uneven plasticizaed morphology, and a large amplitude (49.0 μm) setting can obtain a more uniform melt, as shown in Figure 23. Michaeli et al. [136] have recorded and evaluated the homogenization and plasticization results of molten materials with a microscope, which showed that the materials had a regular and homogeneous crystalline structure.

It can be seen that the ultrasonic amplitude, as one of the important process parameters, has a significant impact on the plasticizing ability and quality. However, due to the lack of strong shear force field caused by the traditional screw plasticizing process, UPMIM does not have good material mixing ability.

In the developed Configuration 2 later, the sonotrode is flush with the gate, the concentrated energy is used to plasticize the polymer, and then injection filling is performed. A new application mode of ultrasonic waves, including continuous ultrasound and intermittent ultrasound, is considered as one of the parameters [106]. Optimal plasticizing results are achieved using a medium setting. However, results showed the different molecular weight distribution in three regions. In the first few seconds of ultrasonic processing, the material closer to the sonotrode is exposed to ultrasonic energy and generates heat earlier, which is the first and most easily degradable. However, the polymer far away from the probe can only receive attenuated ultrasonic energy during the same exposure time, which is a challenge to the plasticization uniformity. At the same time, the limited plasticizing capacity has become one of the factors restricting its large-scale mass production.

**Figure 24:**
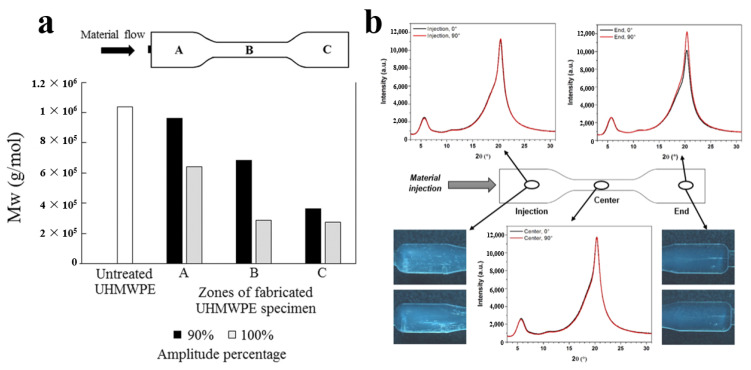
(**a**) The molecular weights of three different regions of the molded sample under different ultrasonic amplitudes [106]; (**b**) SWAXS measurements collected in PA12 sample at the injection, center, and end regions by considering patterns at 0° and 90° [108].

Grabalosa et al. [108] found that due to the application of ultrasonic energy, the polymer sample realized linear flow from the middle to the end, resulting in a better appearance, as shown in Figure 24a. The short distance (2 mm) from the plasticizing chamber to the mold cavity is insufficient to make the melt flow uniform. The chain arrangement is mainly due to the formability of polymer at the exit of the mold during the injection. The arrangement of PA12 chains causes the end sample region to be different from the injection and center regions, and the SWAXS measurements show different absorption, as shown in Figure 24b.

It can be seen that the molecular weight distribution is not uniform along the radial direction centered on the plasticizing chamber; i.e., there are differences in the plasticizing effect in the whole molding process. An uneven distribution of molecular weight may lead to residual stress and then affect the precision of injection parts. Therefore, this defect can be avoided by limiting the size of the molded part.

#### 3.3.8. Degradation Problem

Most polymer materials are suitable for mature conventional micro-injection-molding processing, but there are no relevant standards for UPMIM. High-quality parts can only be obtained under the coordination of various parameters. If the ultrasonic energy is relatively low, only partial interfaces are welded together [137], and excessive ultrasonic energy can cause polymer degradation; the most intuitive is the change in topography. 

**Figure 25:**
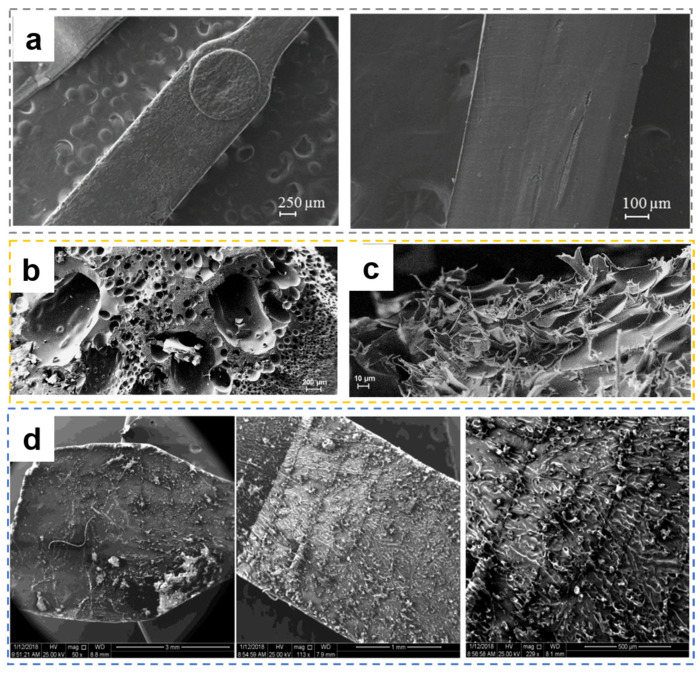
(**a**) A PLA specimen molded at 24-300-1.2 condition (please refer to the original text for detailed explanation) without any physical defects [115]; (**b**) Sprue with numerous holes from 50 μm to 1 mm; (**c**) Molten material remained adhered to the sonotrode sufface [112]; (**d**) Highly degraded sample with very low tensile strength [113].

The morphology of specimens was evaluated by scanning electron microscope (SEM) in many studies. Sacristán et al. [112] evaluated PLA samples under different processing conditions. The SEM micrographs of the processed PLA sample under optimal parameters are shown in Figure 25a, in which no obvious physical defects can be detected [115]. Numerous holes from 50 μm to 1 mm will occur in the samples when a higher molding pressure (3 bar) and a lower ultrasonic amplitude (28.4 μm) are applied, as shown in Figure 25b, which can be explained by the cavitation process. The rough morphology of the material adhered to the surface of the sonotrode also indicates the occurrence of degradation during plasticization, as shown in Figure 25c. The above reports illustrate that various process parameters should be set according to different material characteristics in UPMIM, because the incompatible parameters with material properties not only affect the molding quality but also the physical and chemical properties of injection parts.

The experimental results of Dorf et al. [113] proved that the samples without pores and visual dark marks (without degradation) show the highest tensile strength, while the highly degraded samples have very low tensile strength in other sets of parameters, such as low plunger speed and long ultrasonic exposure time. A high degradation of polymer material can be observed on the sample shown in Figure 25d. Another manifestation of degradation is a decrease in molecular weight. For example, under the condition that the average molecular weight is only reduced by less than 6%, PLA and PBS can be molded in powder form [54]. The main processing parameters in UPMIM are the amplitude of the vibration, the sonicating time, and the applied force [105]. Therefore, there are significant differences between MIM and UPMIM processes in parameter control, process control, and parameter scale, which indicates that it is necessary to improve the understanding of the process [138].

Table 4 lists some of the best parameters for ultra-high molecular weight polyethylene (UPMIM) without degradation. Note that degradation always happened in parts molded by UHMWPE via ultrasonic processing, but the final thermal stability was not significantly influenced by a decrease in the molecular weight [106], and the thermal stability of all the UHMWPE/graphite composites was considerably better than that of pure UHMWPE [114]. The prepared specimens showed considerably better mechanical properties than pure UHMWPE.

### 3.4. Theoretical Interpretations

Theoretical research about UPMIM is currently lacking, mainly focusing on ultrasonic energy balance and heat generation mechanisms, including frictional heating [110,111,140] and viscoelastic heating [20,55]. Up to now, although the heat mechanism of ultrasonic welding has been reported [141,142,143,144], the research on the thermal mechanism of ultrasonic plasticization of polymer still needs further improvement.

#### 3.4.1. Ultrasonic Energy Balance

Grabalosa et al. [108] proposed a mathematical modeling method based on acoustic/ultrasonic energy balance, including theoretical dissipated energy, energy provided by the process, and energy required for melting materials. According to Rienstra and Hirschberg [145], Equation (7) describes the acoustic energy of a homentropic flow, which is given as:

(7) $$\frac{\partial }{\partial t}\left(\rho e+\frac{1}{2}\rho v^{2}\right)+\nabla \cdot \left[v\left(\rho e+\frac{1}{2}\rho v^{2}+p\right)\right]=-\nabla \cdot q+\nabla \cdot \left(\tau \cdot v\right)+f\cdot v$$

where *ρ* is the density of the material, *e* is the internal energy per unit mass, *q* is the heat flux resulting from the heat conduction, *v* is the material’s flow velocity, *f* is the external force density, *p* is the pressure, *τ* is the viscous stress tensor, and ∇ is the symbol representing the gradient operator.

In terms of the process, it is considered the dissipated energy resulting from oscillation movement and the movement of the sonotrode. The dissipated heat flux during the ultrasound injection process could be found from the following expression (8):

(8) $${\dot{Q}}_{\mathrm{avg}}=4pa\omega$$

where *ω* is the ultrasonic frequency, and *a* is the oscillatory amplitude of the sonotrode tip. 

Whereas, in terms of the material, the theoretical melting energy required is also included. Considering the amount of material that is melted in each cycle (the heat capacity of the material and the fusion heat) as well as the temperature increase required to reach the material’s melting temperature, an approach of the minimum energy required can be obtained using the following Equation (9):

(9) $$Q_{m}=mC_{p}\Delta T+m\Delta H_{f}$$

where *C_p_* is a heat constant, ∆*T* is the temperature difference, and ∆*H_f_* represents the enthalpy fusion.

*C_p_* = 2.10 J/gK and ∆*H_f_* = 245 J/g were chosen when processing 300 mg of polyamide with different processing parameters. Results from the theoretical approach indicate that the power delivered by the sonotrode is lower than the power required to melt the material in 1 s, which explains why it was not possible to obtain completed parts with such vibration time, in accordance with the experimental observation.

#### 3.4.2. Heating in Solids

##### Friction Heating

One of the main heat sources in the initial stage of ultrasonic plasticizing polymer particles is interfacial friction heating. According to our previous research referring to ultrasonic welding [146,147], interfacial friction heating has a significant influence on subsequent viscoelastic heating [20,110,111]. Interfacial friction between polymer granulates is dry friction type, the interfacial friction heating is mainly contributed by sliding friction [111], and the heating rate at the interface between two granulates can be described as:

(10) $$Q\left(t\right)=\vec{\tau \left(t\right)}\times \vec{v\left(t\right)}$$

where $\vec{\tau \left(t\right)}$ is the equivalent friction stress, and $\vec{v\left(t\right)}$ is the relative sliding velocity. 

The relative movement between polymer granulates causes frictional heating. The relative sliding rate and equivalent friction stress, which are closely related to the heat flow rate, increase with the increased ultrasonic amplitude [110]. The main parameters affecting the friction properties of polymers include contact pressure, velocity, and temperature [148].

Most research papers are about the effect of parameters including processing parameters and structural parameters on the heating mechanism during ultrasonic plasticizing. In terms of processing parameters, studies of the ultrasonic amplitude, frequency, and pressure on the heating rate of polymers have been carried out [20,55,107,110,111,149]. 

**Figure 26:**
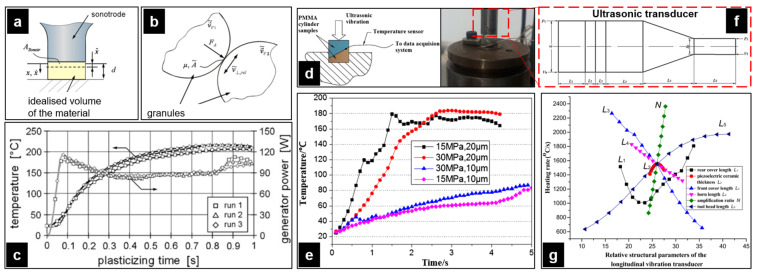
(**a**) Viscoelastic heating and (**b**) friction heating in UPMIM; (**c**) Example for temperature courses during plasticizing for three runs at equal parameter settings [107]; (**d**) Schematic and experimental setup for interfacial friction heating; (**e**) Interfacial friction heating under different plasticization pressure and amplitude [110]; (**f**) Longitudinal vibration transducer structure; L_1_—rear cover length, L_2_—piezoelectric ceramic thickness, L_3_—front cover length, L_4_—horn length, L_5_—ultrasonic sonotrode length, S_1_—large cross-section area of horn, S_2_—small cross-section area of horn, F_1_—rear cover force, V_b_—rear cover vibration velocity, F_2_—front cover force, V_f_—front cover vibration velocity, amplification ratio N = (S_1_/S_2_)^−2^; (**g**) Influence of the structural parameters on the heating rate [149].

The process of the temperature curve is the typical process of all tested polymers up to now, which is the same as the method proposed by Michael et al. [107], as shown in Figure 26a,b. The first tenth of a second of the process cycle is the stage of rapid heating, which can be explained as the effect of rapid friction heating. It has been demonstrated that there is friction heating only at the initial stage of the plasticizing process in the study of Wu et al. [110], where the interface could have a steep temperature increase up to polymers flow temperature in 0.078 s in the case of PMMA granulates. The similar curve trends in Figure 26c,e show that the heating rate decreases from a certain point until the melting temperature level is reached. In Figure 26e, the ultrasonic amplitude was confirmed to have more significant impact than the plasticizing pressure on the interfacial friction heating. Since the energy of the ultrasonic wave is proportional to the square of the amplitude, it is necessary to amplify the amplitude through the booster in order to obtain the ultrasonic wave with a large energy. When ultrasonic amplitude is increased from 10 to 30 μm, the average heating rate is increased from 460.4 to 1687.5 °C/s, which leads to the ultrasonic plasticization of polymer particles from 30 to 160 °C.

For granular materials, ultrasonic amplitude is a more significant factor than plasticizing pressure on interfacial friction heating. However, many uncertain factors are introduced due to the compressibility of granular materials, which make the plasticizing quality and injection speed unstable: that is, it is not conducive to the process stability. However, it seems that conventional rod materials can improve the process stability; hence, the heating principle and mechanism need to be further developed.

However, in actual experiments, the temperature sensor may only be damaged after several tests, resulting in no measurement curve in this case [110]. Based on the repeatability of the experiment, the heat generation rate detection can be determined using sapphire windows [105,129,150] or high-speed infrared cameras [55,105]. Janer et al. [55] studied the polypropylene heating when applying high-power mechanical ultrasound with a high-velocity infrared camera. The results show that the heating of a polypropylene cylinder caused by ultrasonic vibration is highly uneven, and there are different heating steps in this process. The development of new thermal detection technology plays an important role in understanding the plasticization process. Coordinating different plasticizing heating stages and injection speeds will become an important step in process development.

In the aspect of structural parameters, the ultrasonic plasticization of polymer particles was studied by introducing different combinations of structural parameters of transducers and the interaction mechanism of friction heating and plasticization of polymer particles under the longitudinal vibration excitation. The friction plasticizing heating equations of the polymer granulates under the longitudinal vibration excitation were established by Li et al. [149]. Figure 26f shows the longitudinal vibration transducer structure used in UPMIM. The analysis results show that in the initial stage of ultrasonic plasticization, among the structural parameters of the longitudinal vibration transducer, the magnification of the horn has the greatest effect on the heating rate of frictional plasticization. In addition, the front cover length, ultrasonic sonotrode length, and the horn length have little influence on the heating rate, while the piezoelectric ceramic thickness of the piezoelectric ceramic and the length of the rear cover have the least influence on the heating rate.

**Figure 27:**
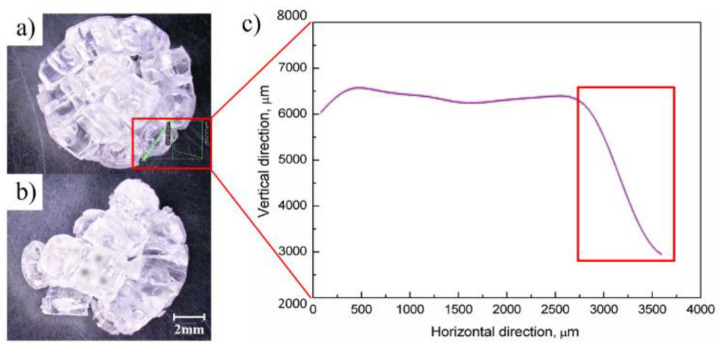
The measurement process (**a**,**b**) and results (**c**) of friction angle [140].

Additionally, Jiang et al. [140] characterized the contact angle of some plastic polymer pellets (PMMA, PP, and PA66) with a super-high magnification lens zoom 3D microscope, taking into account the random stack of materials and extremely short interfacial friction heating time compared with the certain contact area of ultrasonic welding [151]. With the increasing parameter level, the proportion of interfacial friction angle in the range of 0°–10° and 80°–90° increased, while the proportion in the range of 30°–60° decreased accordingly, as shown in Figure 27. In the actual production process, the distribution of the contact angle is affected by factors such as particle size and shape. Therefore, the uncertain factors introduced by the particle material are not conducive to the stability of the UPMIM. The regularization of production materials may become one of the important development directions of this process.

**Figure 28:**
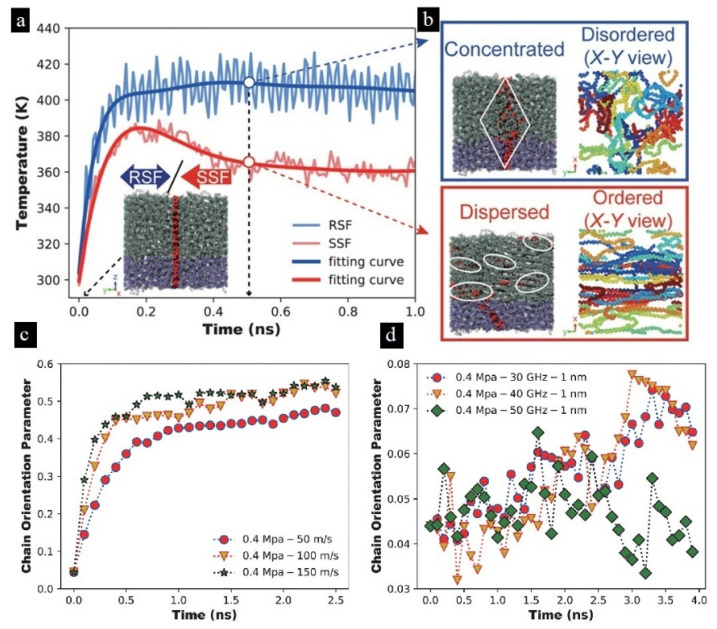
(**a**) Changes in temperature as a function of time in SSF and RSF modes; (**b**) Friction simulation results of PE internal structure under two friction modes at the same simulation time point; Variation of chain orientation parameters with sliding time in the SSF process (**c**) and the RSF process (**d**) [152].

Subsequently, Wu et al. [152] performed united-atom molecular dynamics simulations to reveal the interfacial friction heating mechanism of amorphous polyethylene under single sliding friction (SSF) and reciprocating sliding friction (RSF) modes. The results show that RSF is a more efficient way of generating heat than SSF in terms of heating as shown in Figure 28a; that is, ultrasonic plasticization is the one with higher heating efficiency. Figure 28b shows the simulation results of the molecular chain orientation in the two friction models at the same time point. In terms of orientation, the molecular chain in the RSF model is disordered due to the frictional form of high-frequency vibration restricted in its region, as shown in Figure 28d. Therefore, the concentrated high-frequency chain motion related to molecular rearrangement is considered as the main mechanism for enhancing frictional heating at the RSF interface.

**Figure 29:**
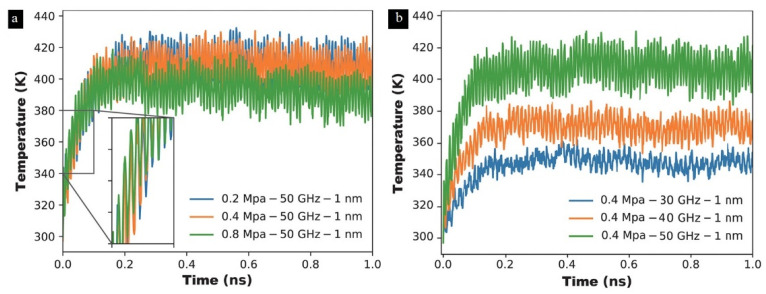
Variation of PE bulk temperature with time in RSF mode under (**a**) various loading pressures and (**b**) various frequencies [152].

Secondly, as far as process parameters are concerned, the effect of sliding rate on temperature rise is more critical than that of loading pressure, as shown in Figure 29. This work illustrates the advantages of the ultrasonic plasticization principle compared to screw plasticization. As a potential tool, molecular dynamics simulation is indispensable for deepening process understanding.

##### Viscoelastic Heating

**Figure 30:**
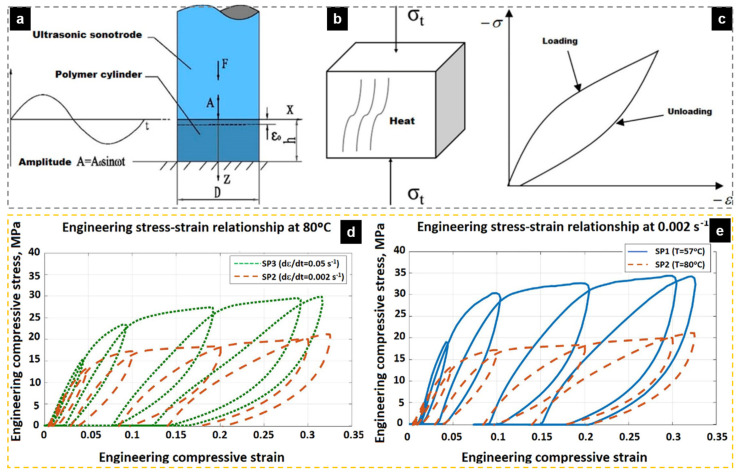
(**a**) Simplified loading conditions for polymer pellet; (**b**) Simplified viscoelastic heating model in ultrasonic plasticizing; (**c**) Typical stress–strain curve of polymer material in a vibration cycle [20]; (**d**,**e**) Stress–strain curves obtained from the cyclic compression tests of three cylindrical specimens (SP1, SP2, and SP3) of PP. The tests were performed by the Janer et al. with different processing conditions [155].

The viscoelastic heat generation effect is a “self-heating process”. It is well known that polymeric materials are sensitive to strain rate and temperature. When polymer particles are subjected to a vibration pressure load, due to the hindrance of the internal macromolecular segments, the segments generate “internal friction” during the deformation and recovery process. The mechanical work performed by external forces is converted into the heat of the polymer itself, causing an increase in the local temperature of the polymer [153], which is both strain and strain-rate dependent [154]; the compression and unloading curves do not coincide, as shown in Figure 30d, forming a “hysteresis loop” whose area is equal to the thermal energy increase of the polymer in a single vibration cycle. During continuous loading, the polymer consumes mechanical energy in each cycle and is converted into the thermal energy of the polymer. The thermal energy increase of the polymer in unit time under vibration load is:

(11) $$Q=f\oint \sigma \left(t\right)d\varepsilon \left(t\right)=f\oint \sigma \left(t\right)\frac{d\varepsilon \left(t\right)}{dt}dt.$$

When the vibration load is a sinusoidal alternating load, the expression of stress and strain is:

$$\sigma \left(t\right)=\sigma_{0}\sin\left(\omega t\right)\;\varepsilon \left(t\right)=\varepsilon_{0}\sin\left(\omega t-\delta \right)$$

where *σ*_0_ is the stress amplitude, *ε*_0_ is the corresponding strain amplitude, *δ* is the phase angle of the strain hysteretic stress, and *ω* is the angular frequency of the vibration load.

(12) $$Q=f\oint \sigma \left(t\right)d\varepsilon \left(t\right)=f\sigma_{0}\varepsilon_{0}\omega \overset{2\pi /\omega }{\underset{0}{\int }}\sin\omega t\cos\left(\omega t-\delta \right)dt=f\pi \sigma_{0}\varepsilon_{0}\sin\delta$$

For the ultrasonic plasticizing polymer process, the alternating load frequency of polymer particles is in the kilo hertz range, and the amplitude is in the micron order. The stress and strain of the polymer can reach a larger order of magnitude with a smaller amount of plasticization. The viscoelastic heat generation effect is also an important heat generation effect in the ultrasonic plasticization heat generation process.

Additionally, the irregular shape and random stacking of polymer materials are causes of the uneven and complex stress field in UPMIM. Hence, in order to solve this problem, as shown in Figure 30a, the loading conditions of micro-units in polymer pellets are simplified. It is assumed that the micro-unit cell is loaded with ideal uniaxial normal stress *σ*(*t*), which is a sine function with the same frequency as the ultrasonic vibration. The diameter of the polymer body cylinder is 10 mm, the height is 5 mm, and it is periodically loaded by the sonotrode, as shown in Figure 30a [20].

In order to illustrate the complex thermomechanical behavior of PP, Janer et al. [155] applied cycles of loading and unloading in uniaxial compression and incremental levels of loading on cylindrical specimens (diameter = 12 mm; height = 20 mm) of polypropylene. The obtained curves, as shown in Figure 30d,e, were tested by a 5 kN *MTS Landmark^®^* servohydraulic machine, with several combinations of temperature and strain rate. 

**Figure 31:**
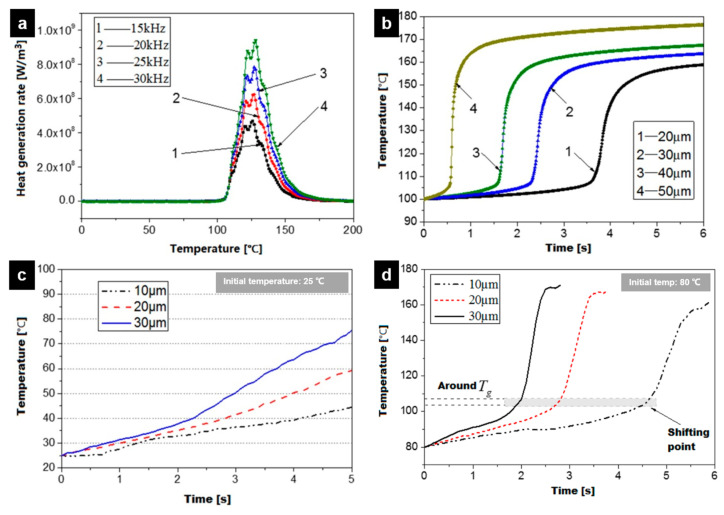
(**a**) Viscoelastic heat generation rates under different ultrasonic frequencies; (**b**) Viscoelastic heating curve of PMMA at various ultrasonic amplitudes, when the initial temperature is 25 °C (**c**) or 80 °C (**d**) [20].

Based on the generalized Maxwell model, Arrhenius model, and semi-empirical Williams Landel Ferry (WLF) model, the literature has involved the study of the viscoelastic heat generation mechanism in the process of ultrasonic welding through theoretical modeling and experimental research [141,156,157,158]. In UPMIM, processing parameters such as ultrasonic frequency, amplitude, and initial temperature were considered in the viscoelastic heating study by Wu et al. [20], as shown in Figure 31. The results show that ultrasonic amplitude is a more effective factor than ultrasonic frequency in affecting the heat generation rate. As far as the initial temperature of the material is concerned, the initial temperature of the PMMA cylinder has no significant effect on the viscoelastic heating rate before reaching 105 °C.

In ultrasonic machining, the hammering phenomenon caused by periodic contact loss caused by high-frequency vibration between an ultrasonic sonotrode and adherents directly affects heating efficiency, but there is no relevant research in UPMIM. 

Viscoelastic heating, which is maximized around the glass transition temperature of the thermoplastic polymer, can be quantified based on the work by Tolunay et al. [159].

(13) $${\dot{Q}}_{bulk}=e\frac{\omega \varepsilon^{2}E^{\prime \prime }}{2}$$

where *e* is called the hammering efficiency, i.e., the ratio between actual heat generation (with hammering) and ideal heat generation (without hammering), *ω =* 2π*f* is the pulsation of vibration, $E^{\prime \prime }$ is the loss modulus of the material, and *ε* is the amplitude strain tensor.

In ultrasonic vibration processing, the sonotrode tip has a displacement that is sinusoidal as shown in:

(14) $$u_{sono}=a_{sono}\cos\left(\omega t\right)$$

where *α_sono_* is the amplitude, *ω* = 2π*f* is the pulsation, and *f* is the frequency.

**Figure 32:**
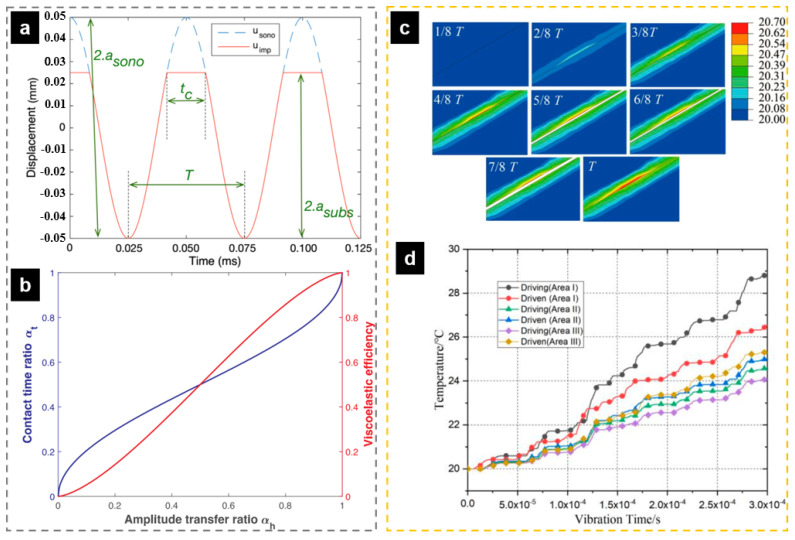
(**a**) Displacement of the sonotrode and upper adherend versus time; (**b**) Viscoelastic efficiency *e* vs. amplitude transfer ratio *α_h_* [144]; (**c**) The nephogram of temperature change of analysis results during T/8–T; (**d**) Transient temperature trend curve of the node with the highest temperature in the three regions of the friction interface [111].

As a result of the hammering effect and the loss of contact between the sonotrode and the composite, the imposed displacement *μ_imp_* on the top surface of the top adherend is not equal to *μ_sono_*. Rather, it is a truncated sine, as illustrated in Figure 32a. The contact time ratio can be defined as:

(15) $$\alpha_{t}=1-\frac{t_{c}}{T}$$

The *t*_c_ is the loss of contact time during an ultrasonic period *T =* 2π/*ω*. *α_t_* ranges between 0 and 1 and reaches 1 for a perfect contact with no hammering. Thus, the viscoelastic efficiency *e* of the process is:

(16) $$e=\alpha_{t}+\frac{\sin\left(2\pi \alpha_{t}\right)}{2\pi }$$

which can be expressed as a function of the amplitude transfer ratio *α_h_* using the following Equation (15). Figure 32b shows the dependency of the efficiency *e* versus the amplitude transfer ratio *α_h_*.

(17) $$e\approx \alpha_{h}$$

In the UPMIM, Peng et al. [111] found that temperature increases occurred only in the loading stage (NT~(2*n* + 1)T/2, N = 0, 1, 2,…). In the unloading stage ((2N + 1)T/2~(N + 1)T, N = 0, 1, 2,…), the temperature of the friction surface will be transferred with the form of heat conduction in the polymer friction interface, as shown in Figure 32c,d. In the unloading stage, the friction surface temperature is almost constant due to the short heat transfer time and low heat transfer coefficient, which means that the ultrasonic hammer effect directly has a significant impact on the heating rate.

## 4. Conclusions and Remarks

In order to address the challenges confronted by the micro-injection-molding process, the application of power ultrasound has proved to be a successful and promising attempt for various polymeric micro-molded parts. In the case of the macro components with a surface with micro/nano functional structures, ultrasonic-assisted micro-injection molding (UAMIM) has been developed to facilitate the polymer melt flow in micro/nano cavities. This could improve the replication fidelity, avoid the use of high injection speed/pressure, and accordingly reduce the energy consumption. In addition, the ultrasonic vibration is also beneficial for the movement of the macromolecules and therefore has the possibility to tailor the micro morphologies (orientation, crystallization, etc.) and molding defects such as weld line for improved molding quality. On the other hand, for the small components with weight in milligram scale, ultrasonic plasticization micro-injection molding (UPMIM) has been developed to address the challenge regarding the excessive plasticization. Ultrasonic vibration energy could be used as the only source for the plasticization of the plastic raw materials with just the amount needed for the successive molding. Furthermore, with the unique simultaneous plasticizing and injection, the polymer melt could be immediately injected into the mold cavity after plasticization, which is essential to reduce the residence time for thermally sensitive plastics. Therefore, the material utilization could be improved via UPMIM besides the benefits of the power ultrasound demonstrated in the UAMIM.

This review provided a general and introductory overview of the application status of power ultrasound in both UAMIM and UPMIM from the aspects of scale and size effect, process principles, configuration design, engineering characteristics, and theoretical interpretations. In the case of UAMIM, the power ultrasound is used as an external auxiliary energy field. The research is focusing on the influence mechanism of the power ultrasound on the polymer melt flow properties and the micro morphological evolution. However, in UPMIM, the power ultrasound becomes the dominant energy source for the plasticization and injection. The research focus is extended to the new plasticization concept via ultrasonic vibration and to the mechanism of the possible change of the material properties. So far, the tuning power ultrasound for enhanced MIM performance of thermoplastic polymers is still challenged by several issues such as the melt flow behavior in the micro-cavity in the presence of the ultrasonic vibration in UAMIM, the stability of the power ultrasound system under the coupled loading conditions during the unique simultaneous plasticization and injection in UPMIM, and the reproducibility of the molding quality in both technologies.

In summary, the instructive suggestions for ultrasonic energy field still need to be further studied and standardized more systematically for UAMIM, including but not limited to the different application methods (direct or indirect), the setting of the vibration point (parallel or perpendicular), and the energy utilization efficiency. For UPMIM, the prerequisites for reduced energy consumption and the significance of improved filling and molding ability need to be further clarified. In addition, the stability improvement of the system, process, and parts quality is still facing challenges. What may be predicted is that the application of power ultrasound in the MIM will become even more reliable, accurate, and versatile in the future.

## Figures and Tables

**Table 1:** List of the current schemes of ultrasound action. In this table, only the basic setting is listed; for detailed configurations and process parameters, please refer to the source.

Vibration Point/Region	Direction	Indirect/Direct	Reference
Gate	Vertical	Direct	[18,48]
Middle of mold insert	Vertical	Indirect	[45,49]
		Direct	[31,38,46]
Integrated with mold	Parallel	Indirect	[40,43,50]
Whole of the mold	Vertical	Direct	[51]

**Table 2:** Comparison of the best reproduction quality and process parameters between *Μ*uvim (UAMIM) and *μ*ICM [38].

Processes	Mold Temperature [°C]	Injection Speed [mm/s]	Packing Pressure [MPa]	Production Cycle [s]	Height [μm]	Standard Deviation
*μ*UVIM (UAMIM)	70	102	123	11.86	36.46	0.75
*μ*ICM (VMV + RHCM)	108	131	150	45	31.12	0.73

**Table 3:** Examples processed by UAMIM.

Molded Parts		Material Trade Name	MIM Machine and Parameter	Ultrasound Parameter	Results	Ref.
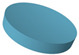	Concave Lens	Polycarbonate (PC) TARFLON A2200	AZ700 Compression force: 980 kN Cooling time: 120 s	f = 19 kHz Amplitude: 0–11 μm Oscillation: 0–60 s	Replication properties ↑ Residual optical strain ↓	[18]
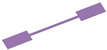	Micro-Tensile Sample	Polypropylene (PP) PPH 734-52 RNA	ARBURG Allrounder 320C Clamping force: 600 kN	f: 20 kHz Max. power: 800 W Max. amplitude: 11 μm	Weld line strength ↑	[40]
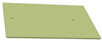	A Flat Sample	Polycarbonate (PC) Teijin AD5503	ARBURG Allrounder 320C Clamping force: 600 kN	f: 19 kHz Max. power: 2 kW Max. amplitude: 15 μm	Pressure loss ↓ Residual stress ↓ Filling efficiency ↑ Thickness of the frozen layer ↓	[31,39]
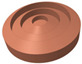	Fresnel Lenses	COC Zeonex 480R	FANUC ROBOT S-2000i 50B	f: 27 kHz	Filling performance ↑ Symmetric deviation ↑	[45]
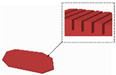	Microchannel Array	PMMA HT50Y	DQ-1500T-A Clamping force: 15,000 kN	f: 28 kHz Max. amplitude: 10 μm	Average weight/height ↑ standard deviation ↑ replication rate ↑	[38]
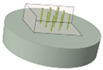	Micro-Needle Array	Polymethylmethacrylate (PMMA) JPC Novatec BC06N	FANUC ROBOT S-2000i 50B	f: 55 kHz Max. amplitude: 28 μm	Filling rate ↑ Material properties ↑	[49]

**Table 4:** Optimized parameters for filling without degradation.

Material	Geometry Tensile Test [cm^3^]	Standard	Amplitude [μm]	Time [s]	Pressure/Velocity	Ref.
PLA	1.5 × 0.1 × 0.1	IRAM-IAS-U500-102/3	48.1	3	3 bar	[112]
PLA Poly (nonamethylene azelate) (PE99)	1.5 × 0.1 × 0.1	IRAM-IAS-U500-102/3	24	1.2	300 N	[121]
PA12	33 × 2.5 × 1.25	ASTMD638	35	5	2 bar	[108]
Poly (ε-caprolactone) Graphistrength^®^ C10 carbon nanotubes	1.5 × 0.1 × 0.1	IRAM-IAS-U500-102/3	37	7 or 8	2500 N	[139]
PEEK	30 × 2 × 2	ASTMD638	52.2/58	8/5	5/6	[117]
PPSU	30 × 2 × 2	ASTMD638	58/40.6	1.4/2.8	11/5	[113]
UHMWPE	30 × 2 × 1	ISO-527-4	50.6/56.2	—	—	[106]
UHMWPE + graphite				—	—	[114]

## Data Availability

Not applicable.
